# Multi-Horizon Probabilistic Wind Power Forecasting for Mountainous Wind Farms Based on Entropy-Weighted Fusion and Permutation Entropy-Guided Decomposition

**DOI:** 10.3390/e28080902

**Published:** 2026-08-10

**Authors:** Chunhui Liu, Bilin Shao, Dawen Nie, Ning Tian, Hongbin Dai, Huibin Zeng, Wei Zhao, Xue Zhao, Xinyu Liu, Caiyun Qin

**Affiliations:** 1 School of Management, Xi’an University of Architecture and Technology, Xi’an 710055, China; lchunhui1806@xauat.edu.cn (C.L.); sblin@xauat.edu.cn (B.S.); ndw1999@xauat.edu.cn (D.N.); tning4891@xauat.edu.cn (N.T.); zhaowei1806@xauat.edu.cn (W.Z.); zhaoxue1018@xauat.edu.cn (X.Z.); lxiny0119@163.com (X.L.); qincaiyun@xauat.edu.cn (C.Q.); 2 College of Artificial Intelligence and Advanced Interdisciplinary Sciences, Chongqing Normal University, Chongqing 401331, China; zenghuibin@cqnu.edu.cn; 3 Ningxia Water Group Yunlian Technology Co., Ltd., Yinchuan 750001, China

**Keywords:** probabilistic wind power forecasting, entropy-weighted fusion, permutation entropy, signal decomposition, natural gradient boosting

## Abstract

Wind power integration into mountainous power grids amplifies probabilistic forecasting challenges arising from strong non-stationarity, multi-source meteorological redundancy and frequent curtailment events. To address the limitations of existing approaches, this paper proposes a multi-horizon probabilistic forecasting framework integrating multi-perspective entropy-weighted fusion, permutation-entropy-guided decomposition, and residual-anchored probability modelling. First, an Entropy-Weighted Multi-criteria Permutation Feature Importance (EW-MPFI) module fuses KSG mutual information, Tree-SHAP, and elastic-net permutation importance through entropy-based weighted aggregation, distilling 23-dimensional meteorological inputs into eight informative features while suppressing single-criterion bias. Then, a three-stage decomposition strategy applies ICEEMDAN primary decomposition, permutation-entropy and sample-entropy guided band reconstruction, and SSA secondary refinement on high-frequency components, achieving complexity-aligned multi-scale separation. Finally, a decomposition-aware patch-based Transformer backbone (DPC-Former) generates three-quantile point forecasts, upon which an NGBoost residual layer models the conditional distribution via natural-gradient optimization in the information-geometric parameter space. Case studies on a 130 MW mountainous wind farm in Sichuan, China, covering 8736 15-min samples with 566 curtailment samples (6.48% of the dataset), show that, under the partition-wise offline batch-evaluation protocol, the proposed framework achieves an NMAE of 5.21%, an NCRPS of 3.74%, and a PICP80 of 0.84 across forecasting horizons from 15 min to 4 h. Ablation analysis attributes NMAE improvements of 25.36% and 24.57% to the decomposition and feature-selection modules, respectively, while 50-seed ensembling further reduces NCRPS, NMAE, and NRMSE by 7.40%, 7.00%, and 13.70% relative to single-seed training. A fixed-checkpoint test-block diagnostic further shows limited sensitivity at approximately weekly and three-day decomposition cadences, but a material degradation at a one-day cadence. The reported metrics should therefore be interpreted as offline best-case results rather than as performance under strictly causal real-time deployment.

## 1. Introduction

The accelerating low-carbon transition of the global energy mix has elevated wind power from a supplementary energy source to a core pillar of the new-type power system. China has long led the world in installed wind capacity, and as wind resource development on the northern plains approaches saturation, the focus of new deployment is gradually shifting toward complex terrains such as mountainous and hilly regions [1]. In this context, high-accuracy wind power forecasting for mountainous wind farms is of considerable importance for ensuring secure and stable grid operation and for improving the accommodation of renewable energy [2]. Wind power in mountainous areas, however, exhibits far stronger non-stationarity and uncertainty than that in plain-area wind farms owing to the combined effects of complex terrain and meteorological conditions. Achieving both forecasting accuracy and reliable uncertainty quantification across multiple time scales [3], therefore, remains a key bottleneck for the efficient grid integration of mountainous wind power.

Conventional wind power forecasting methods often fail to generalize to mountainous wind farms over complex terrain, where both accuracy and reliability degrade markedly. On the one hand, the strong non-stationarity and multi-scale fluctuations of mountainous wind power exceed the representational capacity of conventional approaches, producing larger short-term forecasting errors and undermining the precision of grid dispatch [4]. On the other hand, mountainous wind power is affected by multi-source uncertainties that conventional point forecasting cannot translate into credible intervals, driving up both dispatch risk and reserve cost under high wind penetration [5]. In addition, existing methods remain limited in selecting and exploiting key features, and fail to fully extract the critical information governing mountainous wind power, which further constrains improvements in forecasting accuracy [6]. Taken together, existing methods fall short in accurate power characterization, key feature selection, and uncertainty quantification, and thus cannot adequately support the high-quality grid integration and practical engineering application of mountainous wind power across multiple time scales.

Driven by complex terrain and meteorological forcing, mountainous wind power exhibits strong non-stationarity and pronounced multi-scale fluctuations, posing compound challenges to forecasting accuracy and reliability. Against this background, four limitations in current research continue to hinder the forecasting performance of mountainous wind power. First, prevailing feature selection schemes mostly rely on a single correlation metric and lack stable fusion across multi-criteria evaluations, resulting in feature rankings that fluctuate substantially across subsets and compromising model reproducibility. Second, prevailing decomposition strategies are typically single- or two-stage and lack complexity-based reconstruction, so that mode mixing persists in high-frequency components and transient mountainous disturbances cannot be adequately separated. Third, prevailing deep learning architectures make no distinction between endogenous power sequences and exogenous meteorological inputs, fail to jointly capture cross-variable interactions and multi-scale local features, and lack dedicated branches for sub-signals at different scales, limiting their ability to characterize power dynamics. Fourth, most probabilistic forecasting methods rely on a single quantile regression or parametric distribution fit for direct output, without a secondary probabilistic layer over the backbone’s predictions or multi-seed ensembling, leaving the uncertainty arising from training stochasticity inadequately characterized.

To address the above limitations, this study develops a multi-time-scale probabilistic power forecasting framework for mountainous wind power, comprising five stages: data preprocessing, feature selection, signal decomposition, a backbone network, and a probabilistic layer. Preprocessing flags curtailment events with a binary mask rather than imputing them. For feature selection, an Entropy-Weighted Multi-criteria Permutation Feature Importance method (EW-MPFI) is proposed, in which KSG mutual information, Tree-SHAP, and elastic-net permutation importance provide three principle complementary scoring perspectives, fused through entropy-based weighting and refined by a Bootstrap stability test that removes fluctuating variables, yielding a robust feature subset that mitigates the ranking instability of any single metric. The signal decomposition stage adopts a three-stage strategy: the power series is first decomposed by ICEEMDAN, the resulting components are grouped into high-, medium-, and low-frequency clusters via K-means based on permutation entropy and sample entropy, and the high-frequency cluster is further refined by SSA, with only the high-frequency band refined to avoid redundant modeling. The backbone, DPC-Former, integrates Cross-Variable Attention (CVA), Multi-scale parallel Encoding (MNE), and a Stability-Gated mixture-of-experts module (StabGate), enabling differentiated modeling of endogenous power sequences and exogenous meteorological inputs. Hyperparameters are tuned via Optuna TPE search, with a duration-weighted pinball loss jointly optimized across multiple scales, and the forward pass outputs multiple quantiles. NGBoost is then trained independently on the backbone outputs at each time scale, learning the parameters of a Gaussian distribution through natural gradients and producing the final probabilistic forecasts via multi-seed ensembling, thereby compensating for the limited distributional expressiveness of single quantile regression and reducing the uncertainty induced by training stochasticity. The proposed framework is validated on field measurements from a mountainous wind farm in Sichuan, China, providing methodological support for the precise dispatch of mountainous wind power and for the accommodation of high-penetration renewable energy in power grids.

The main contributions of this study are summarized in the following three aspects:

(1) A reliable feature selection method tailored to mountainous wind power scenarios is proposed. By integrating multiple principal-complementary feature importance measures with a resampling-based stability test, the method effectively mitigates the ranking instability caused by any single correlation metric, thereby providing a stable and reliable screening mechanism for extracting key information.
(2) A complexity-guided three-stage signal decomposition strategy is designed. The strategy decomposes the power series hierarchically, finely separating multi-scale oscillatory structures while avoiding redundant modeling, thereby alleviating the inadequate multi-scale separation that arises in conventional single- or two-stage decomposition due to conflicting inductive biases across scales.
(3) A multi-time-scale probabilistic forecasting architecture is constructed. The architecture differentially models endogenous power sequences and exogenous meteorological inputs and superimposes probabilistic distribution modeling and multi-seed ensembling upon the deterministic forecasts, thereby compensating for the limited capacity of conventional point forecasting and single quantile regression in expressing uncertainty.

## 2. Related Work

The demand for accuracy and reliability in mountainous wind power forecasting has motivated extensive research. Among the related techniques, anomaly detection and missing-value imputation are relatively mature, and this study therefore adopts a rule-based cleaning procedure rather than a learned detector. Four criteria are applied, one for each anomaly mechanism observed in the raw records: physical range violation, tested against bounds set by the installed capacity and the local climatological range; stuck sensors, identified by a near-zero rolling standard deviation over both a short and a long window; single-point spikes, identified by a Hampel test against the local median; and outliers in the power curve scatter, identified by an interquartile-range test within wind speed bins and applied only to samples not under curtailment. Missing values are then imputed in three tiers according to gap length. The following review summarizes recent progress along four dimensions: feature selection, signal decomposition, deep learning architectures, and probabilistic forecasting.

For feature selection, Pearson and Spearman correlation coefficients and mutual information are computationally lightweight but capture only a single form of dependency, falling short of representing the nonlinear coupling between wind power and meteorological variables [7]. SHAP values and permutation importance offer finer-grained attributions, yet rankings from any single method remain unstable [8]. Existing studies have explored fusing multiple importance metrics, but fusion weights are largely set empirically without stability validation [9], and the chosen metrics often share similar underlying principles, hindering truly cross-perspective complementarity [10].

In signal decomposition, EMD suffers from mode mixing, which is particularly pronounced in the high-frequency band [11]. EEMD and CEEMDAN alleviate mode mixing by injecting auxiliary white noise, yet residual noise and reconstruction bias persist [12]. ICEEMDAN further suppresses such residuals through adaptive noise correction, but it remains a single-stage method that processes all resulting modes through a uniform pipeline [13]. VMD stably separates frequency bands within a variational framework, but it is highly sensitive to the mode number and the penalty factor, and parameter deviations can readily lead to under- or over-decomposition [14]. WPD relies on fixed orthogonal bases for fine-grained band partitioning, but the choice of basis functions and decomposition levels is heavily subjective [15]. SSA, by projecting the trajectory matrix onto principal-component subspaces via singular value decomposition, offers more robust refinement for nonlinear transient oscillations [16].

Nevertheless, the components produced by these single-stage methods differ markedly in complexity, with high-frequency transient disturbances still entangled across multiple components, calling for complexity-based grouping and reconstruction [17]. Existing complexity grouping schemes mostly rely on a single entropy metric, leading to unstable assignment of boundary components [18]. Although reconstruction mitigates redundancy across scales, the fine characterization of high-frequency transient disturbances remains inadequate, motivating the introduction of secondary decomposition. Existing two-stage decomposition schemes, such as CEEMDAN combined with VMD or EMD combined with wavelet packets, typically refine all components uniformly, introducing redundant modeling at medium and low frequencies [19]. High-frequency refinement commonly relies on wavelets or a second pass of VMD, both of which are constrained by basis-function selection and parameter sensitivity [20].

Recurrent networks such as LSTM and TCN capture local patterns well but lack global modeling capacity [21]. Transformers and their variants—including iTransformer and PatchTST—enable long-range modeling and parallel training, yet they treat variable channels coarsely and remain single-scale, failing to jointly address endogenous–exogenous heterogeneity and multi-scale dynamics [22,23]. Subsequent designs introduce cross-variable attention or multi-scale parallel branches, but the former treats channels symmetrically and the latter shares parameters across branches, leaving the primary–secondary distinction between endogenous and exogenous inputs and the frequency-differentiated nature of sub-signals inadequately modeled [24]. Mixture-of-experts architectures can dynamically route experts based on the input, yet their gating typically operates on raw features, leading to sluggish regime-switching response under abrupt meteorological changes [25]. For probabilistic forecasting, parametric distribution fitting presupposes that the power follows a specific distribution family, yet mountainous wind power exhibits multimodal, non-Gaussian behavior, and such rigid assumptions readily induce systematic bias [26]. Quantile regression dispenses with prior distributional assumptions but estimates only discrete quantiles, which are prone to crossing and cannot continuously represent the distributional shape [27]. Kernel density estimation continuously interpolates discrete quantiles into a full probability density, yet it is sensitive to bandwidth choice, with tail estimates showing large variance under small samples [28]. Bayesian neural networks capture epistemic uncertainty via posterior inference over weights, but variational approximation bias and high computational cost hinder engineering deployment [29]. Deep distributional regression has neural networks directly emit distribution parameters, sidestepping the sampling cost of Bayesian inference; however, a single training run produces only one set of outputs, failing to reflect model variability across runs [30].

Drawing on the progress reviewed above, existing methods still face four categories of limitations in mountainous wind power forecasting. At the feature selection level, rankings produced by any single metric remain unstable, and the fusion weights of multi-metric schemes rely on empirical settings. At the signal decomposition level, the components yielded by single-stage methods differ markedly in complexity, while existing complexity grouping schemes depend on a single entropy metric, and secondary decomposition typically refines all components uniformly. At the backbone architecture level, Transformer-based models handle variable channels coarsely and remain single-scale, while mixture-of-experts gating responds sluggishly to regime switching. At the probabilistic forecasting level, parametric distributional assumptions are inconsistent with the multimodal and non-Gaussian nature of mountainous wind power, quantile regression is prone to quantile crossing, and deep distributional regression fails to reflect model variability from a single training run. To address these limitations, this study proposes a probabilistic forecasting framework for mountainous wind power, comprising four core modules. The EW-MPFI module integrates Kraskov–Stögbauer–Grassberger mutual information [31], Tree-SHAP [32], and elastic-net permutation importance [33] as three principal complementary perspectives, fused through objective entropy-based weighting and refined by a Bootstrap stability test [34] that removes variables with large fluctuations. The three-stage signal decomposition strategy builds on ICEEMDAN as the initial decomposition; the resulting components are grouped into high-, medium-, and low-frequency clusters via K-means based on permutation entropy and sample entropy; the high-frequency cluster is then further refined by SSA. The DPC-Former backbone differentially encodes endogenous and exogenous inputs via cross-variable attention, provides dedicated branches for sub-signals of different complexity via multi-scale parallel encoding, and dynamically modulates expert weights according to the operating regime via the stability-gated mixture-of-experts. The NGBoost residual probabilistic layer, applied on top of the quantile outputs of DPC-Former, learns the parameters of a Gaussian distribution through natural gradients and is augmented with multi-seed ensembling to suppress the model variability arising from a single training run.

## 3. Methodology

**Figure 1:**
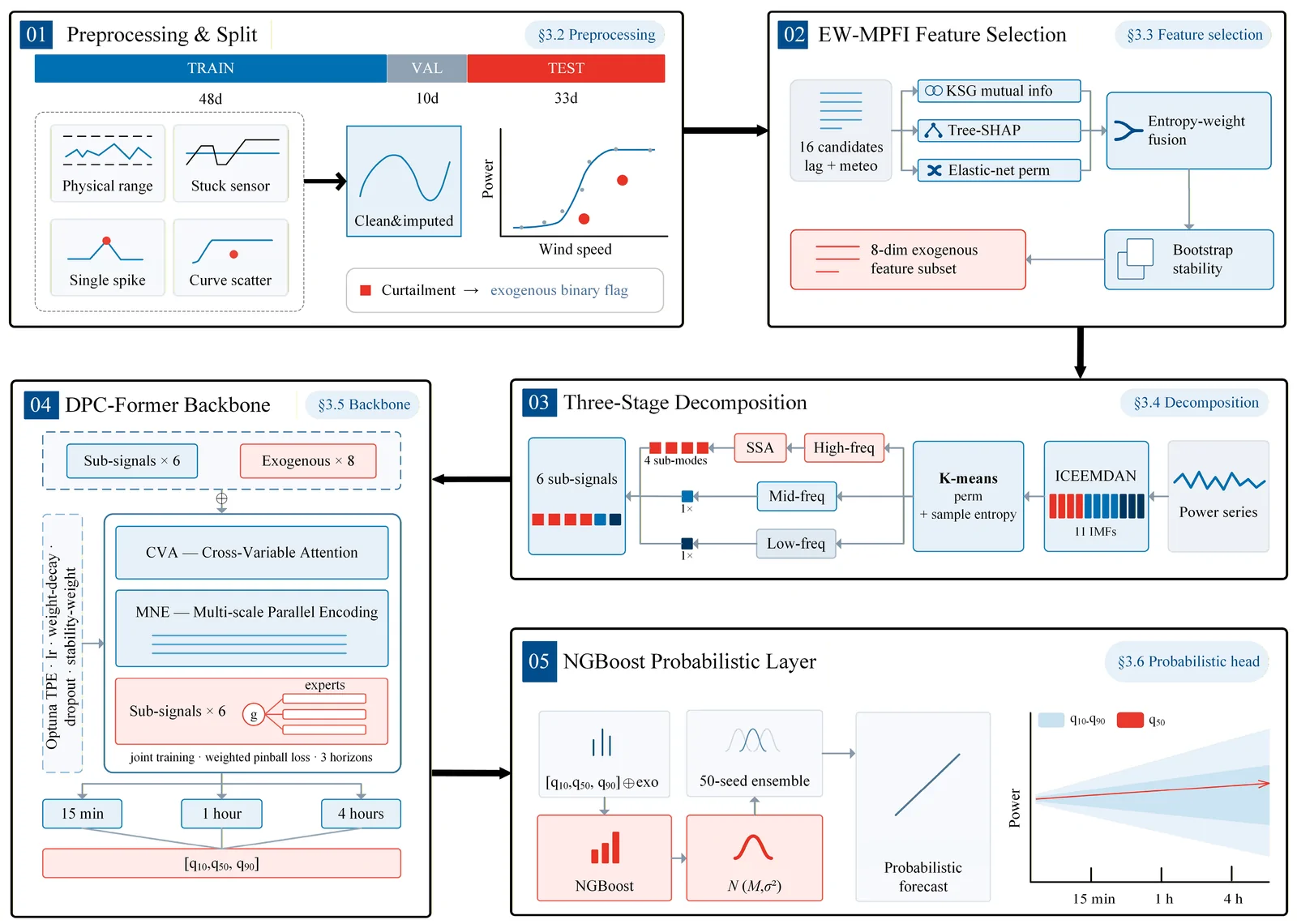
Overall technical pipeline of the multi-horizon probabilistic forecasting framework for mountainous wind farms.

This section presents the unified probabilistic framework for multi-horizon wind power forecasting at mountainous wind farms, organized along the data flow. The overall technical pipeline is illustrated in Figure 1. The framework comprises five stages: data preprocessing and temporal partitioning, EW-MPFI feature selection, three-stage signal decomposition, DPC-Former backbone training, and the NGBoost probabilistic layer.

### 3.1. Overall Technical Pipeline

The framework consists of five sequentially connected stages. The data preprocessing and temporal partitioning stage performs anomaly detection, missing-value imputation, and the splitting of the raw observation series into training, validation, and test sets. The EW-MPFI feature selection stage screens a low-redundancy feature subset from the high-dimensional meteorological candidates. The three-stage signal decomposition stage decomposes the cleaned power series into multi-scale sub-signals. The DPC-Former backbone takes the joint features and sub-signals as input and outputs multi-horizon quantile forecasts. The NGBoost probabilistic layer learns the parameters of a Gaussian distribution on top of the backbone’s quantile outputs to produce the final probabilistic forecasts. The five stages further consolidate into three core methodological modules: EW-MPFI feature selection, the three-stage signal decomposition strategy, and the DPC-Former backbone together with the NGBoost probabilistic layer, which serve as the units of methodological contribution discussed in Section 5 and Section 6. This pipeline is implemented on a spring operational dataset from a mountainous wind farm, and its module-level effectiveness, overall comparative performance, robustness, computational cost, and sensitivity to the test-block decomposition window are systematically evaluated through pipeline and architecture ablations, baseline and probabilistic-method comparisons, multi-seed robustness verification, computational-cost analysis, and an additional decomposition-window diagnostic.

### 3.2. Data Preprocessing and Temporal Partitioning

#### 3.2.1. Anomaly Detection

Anomalous power samples propagate along the data flow and amplify modeling bias in downstream stages. General-purpose methods such as Isolation Forest have been widely applied to energy time series [35], yet they struggle to simultaneously identify four types of anomalies—physical range violations, stuck sensors, single-point spikes, and outliers in the power curve scatter. This section, therefore, designs differentiated criteria A through D tailored to these four mechanisms. Samples flagged by A, B, or D are set to missing and passed to the hierarchical imputation procedure, whereas samples flagged by C are replaced by the local median.

Criterion ***A*** targets physical range violations:

(1) $$M_{A}(t)=I\left[x(t)<x_{\min}\vee x(t)>x_{\max}\right]$$

where *x*(*t*) denotes the value at time *t*, *x*_min_ and *x*_max_ are the lower and upper bounds determined by the installed capacity and the climatological range, and 𝕀[·] is the indicator function, which takes the value 1 when the condition enclosed in the brackets holds and 0 otherwise. Criterion *A*, therefore, returns 1 for a sample lying outside the admissible interval and 0 for a sample within it, so that the criterion acts as a binary flag rather than a continuous anomaly score.

Criterion *B* targets stuck sensors:

(2) $$M_{B}(t)=I\left[\sigma_{w_{1}}(t)<\varepsilon \wedge \sigma_{w_{2}}(t)>\tau_{x}\right]$$

where *σ_w_*(*t*) denotes the rolling standard deviation over a window of length w ending at time t. *w*_1_ and *w*_2_ are the lengths of the short and long windows, set to 4 and 16 steps, respectively. *ε* is a lower threshold close to zero, and *τ_x_* is the minimum effective fluctuation magnitude assigned to variable *x*.

Criterion *C* targets single-point spikes and uses the Hampel filter to detect extreme values that deviate from the local central trend:

(3) $$M_{C}(t)=I\left[\left|x(t)-\tilde{x}(t)\right|>k_{1}\cdot 1.4826\cdot \mathrm{MAD}(t)\right]$$

where $\tilde{x}(t)$ denotes the rolling median over a window of length 4 steps, MAD(*t*) is the median absolute deviation over the same window, 1.4826 is the scaling constant for Gaussian-distribution equivalence, and the threshold factor *k*_1_ is set to 5.

Criterion *D* targets outliers in the power curve scatter. The samples are grouped into bins of the 100 m hub-height wind speed *u*_100_, using a bin width of 0.5 m/s:

(4) $$M_{D}(t)=I\left[P_{\mathrm{actual}}(t)\notin \left[Q_{1}^{\left(b\right)}-k_{2}{\mathrm{IQR}}^{\left(b\right)},\;Q_{3}^{\left(b\right)}+k_{2}{\mathrm{IQR}}^{\left(b\right)}\right]\wedge c(t)=0\right]$$

where *P*_actual_(*t*) is the measured wind power at time *t*, *b* denotes the bin index, *Q*_1_^(*b*)^ and *Q*_3_^(*b*)^ are the first and third quartiles of the power within bin *b*, IQR^(*b*)^ = *Q*_3_^(*b*)^ − *Q*_1_^(*b*)^ is the corresponding interquartile range, *k*_2_ is set to 3, and *c*(*t*) is the curtailment indicator that takes the value 1 during curtailment and 0 otherwise. The criterion includes only samples with *c*(*t*) = 0, thereby excluding curtailment events to avoid false flagging. The 0.5 m/s bin width follows the method of bins of IEC 61400-12-1 [36] and balances resolution against the number of samples per bin: a narrower width leaves the sparse high-wind-speed bins with too few samples for a stable quartile estimate, while a wider one inflates the within-bin spread through the slope of the curve. Because each bin is processed only when sufficiently populated, the criterion is robust to the exact width.

#### 3.2.2. Hierarchical Missing-Value Imputation

Wind power series exhibit strong autocorrelation and a diurnal periodicity, and missing values are therefore imputed in three tiers according to the gap length L [37]. For short gaps with L ≤ 4, linear interpolation is applied:

(5) $$\hat{x}(t)=x(t_{a})+\frac{t-t_{a}}{t_{b}-t_{a}}\left[x(t_{b})-x(t_{a})\right]$$

where *t_a_* and *t_b_* denote the nearest non-missing time points to the left and right of the gap, respectively.

For medium gaps with 4 < L ≤ 16, only meteorological variables are imputed using the mean of the same time-of-day across adjacent days:

(6) $$\hat{x}(t)=\frac{1}{\left|T_{h}\right|}\sum_{s\in T_{h}}x(s),\;T_{h}=\left\{s:h(s)=h(t),\;x(s)\;\mathrm{known}\right\}$$

where *h*(*t*) denotes the 15 min intraday slot index; the power variable is excluded from this tier to prevent target leakage. For long gaps with *L* > 16, bidirectional fill is applied as a fallback.

#### 3.2.3. Temporal Partitioning and Curtailment Mask Generation

The dataset consists of 91 consecutive days of 15-min field measurements from a 130 MW mountainous wind farm in Sichuan, China, collected during spring 2025. The data are partitioned chronologically into training, validation, and test sets of 48, 10, and 33 days, respectively. All partition boundaries are strictly chronological, so that no observation in a later partition precedes any observation in an earlier one. Anomalies are detected by applying the four criteria in sequence—physical range, stuck sensors, single-point spikes, and outliers in the power curve scatter—and imputed through context-aware filling. Curtailment events are flagged rather than imputed, so that the physical reality of dispatch limitation is retained, and the resulting binary mask *c*(*t*) is stored alongside the cleaned series. The mask is not included among the exogenous features supplied to the backbone. It is retained for three purposes: to exclude curtailment periods from the power curve outlier criterion of Section 3.2.1, to identify the segments repaired prior to the decomposition of Section 3.4, and to reweight the training objective in Equation (21). This stage outputs the cleaned power and meteorological series *P*_clean_, which serves as input to the subsequent EW-MPFI feature selection and three-stage signal decomposition, together with the mask *c*(*t*).

### 3.3. EW-MPFI Feature Selection

#### 3.3.1. Multi-Perspective Importance Measures

Let the candidate feature set be 𝓕 = {*f*_1_, *f*_2_, …, *f_p_*} and the target be the wind power *y*. The three perspectives compute mutual information via the KSG estimator, the mean absolute Tree-SHAP value on a LightGBM model, and the increase in mean squared error from random column-wise permutation on an elastic-net regressor [38], respectively. The raw scores are defined as follows:

(7) $$I_{i}=\iint p(f_{i},y)\ln\frac{p(f_{i},y)}{p(f_{i})p(y)}df_{i}dy,\;G_{i}=\frac{1}{N}\sum_{n=1}^{N}|\phi_{i}^{\left(n\right)}|,\;\Pi_{i}=E_{\sigma }[l(y,{\hat{y}}^{\left(\sigma_{i}\right)})-l(y,\hat{y})]$$

where *N* is the number of training samples, *ϕ_i_*^(*n*)^ is the Tree-SHAP value of feature *f_i_* on the *n*-th sample, *σ_i_* denotes the random permutation of the *i*-th column, *ŷ* and *ŷ*^(*σi*)^ are the predictions before and after permutation, and *ℓ* is the mean squared error loss. Negative values of Π*_i_* are clipped to zero. The raw scores from the three perspectives are min-max normalized column-wise to form the score matrix.

#### 3.3.2. Entropy-Weighted Fusion

A single importance measure suffers from methodological bias [39]. This study applies the entropy method to objectively fuse the three heterogeneous measures through weighted aggregation. The information entropy *e_j_* and the corresponding weight *w_j_* of the *j*-th perspective are defined as follows:

(8) $$e_{j}=-\frac{1}{\ln p}\sum_{i=1}^{p}p_{ij}\ln p_{ij},\;w_{j}=\frac{1-e_{j}}{\sum_{k}(1-e_{k})}$$

where *p_ij_* denotes the column-wise probability of the normalized scores, and a higher discrimination yields a larger weight. *C_i_* denotes the composite importance, and the lag group and the meteorological group undergo this procedure independently.

#### 3.3.3. Stability Screening

Bootstrap resampling with replacement is performed *B* times on the training samples, and the full procedure is repeated in each round. The selection frequency of *f_i_* is defined as follows:

(9) $$r_{i}=\frac{1}{B}\sum_{b=1}^{B}I[{\mathrm{rank}}_{b}(C_{i})\leq K]$$

where the truncation threshold *K* is set to the rank at which the cumulative contribution of the composite scores first reaches 90%. A feature is finally selected if its rank is not greater than *K* and its selection frequency, *r_i_*, exceeds the predefined threshold.

Building on *P*_clean_, this section processes the lag power features and the meteorological features as two independent groups, with three principal complementary perspectives applied in parallel to quantify importance: KSG mutual information, Tree-SHAP, and elastic-net permutation importance. These measures are fused through objective entropy-based weighting, after which 50 rounds of Bootstrap stability testing remove variables with large ranking fluctuations. From the 16 candidate variables, an 8-dimensional exogenous feature subset is screened out and used as the exogenous input to the DPC-Former backbone.

### 3.4. Three-Stage Signal Decomposition

#### 3.4.1. ICEEMDAN Decomposition

Curtailment segments are prone to being misidentified as high-frequency oscillations by decomposition algorithms and are therefore linearly interpolated during preprocessing to yield *P*_clean_. This study applies Improved Complete Ensemble Empirical Mode Decomposition with Adaptive Noise ICEEMDAN [40] to perform the primary decomposition of *P*_clean_. Multiple groups of white noise *w*^(*i*)^ are generated in advance, and the IMFs of each noise order are extracted via EMD. The first-order noise IMF is added to the original signal, and the first-order mode is obtained by ensemble averaging through the local mean operator *M*(·):

(10) $${\hat{d}}_{1}=x-\frac{1}{I}\sum_{i=1}^{I}M\left(x+\beta_{0}E_{1}\left(w^{\left(i\right)}\right)\right)$$

where *E_k_*(·) denotes the *k*-th IMF obtained by performing EMD on its input, *I* is the number of ensemble iterations, and *β*_0_ = *ε*·std(*x*) is the noise amplitude coefficient. Subsequent modes are recursively constructed on the previous residual *r_k_*_−1_:

(11) $${\hat{d}}_{k}=r_{k-1}-\frac{1}{I}\sum_{i=1}^{I}M\left(r_{k-1}+\beta_{k-1}E_{k}\left(w^{\left(i\right)}\right)\right),\;k\geq 2$$

where *β_k_*_−1_ = *ε*·std(*r_k_*_−1_). The iteration proceeds until the residual no longer contains a decomposable extremum structure, and the primary decomposition outputs a set of IMFs together with the final residual.

#### 3.4.2. Multivariate Entropy-Weighted Reconstruction

The primary decomposition tends to produce an excessive number of components with strong noise in the high-frequency band. Permutation entropy (PE) and sample entropy (SE) are computed for each component, normalized, and clustered in the resulting two-dimensional space via K-means. The clusters are sorted in descending order of their PE and SE centers and labeled as high-frequency (HF), medium-frequency (MF), and low-frequency (LF) groups [41]. Each group is reconstructed through multivariate entropy weighting, in which pure noise components are assigned low weights to achieve soft denoising:

(12) $$w_{k}=\frac{(1-PE_{k})\cdot N_{g}}{\sum_{j\in g}\left(1-PE_{j}\right)},\;x_{g}^{\mathrm{weighted}}=\sum_{k\in g}w_{k}\cdot c_{k}$$

where *c_k_* denotes the *k*-th component, *g* indexes the group, and the weights are normalized so that the sum of weights within a group equals *N_g_*.

#### 3.4.3. SSA Secondary Decomposition

The high-frequency group carries turbulence-scale components and admits further refinement. This section performs Singular Spectrum Analysis (SSA) as a secondary decomposition on the HF group, which is complementary in inductive bias to empirical-mode or frequency-domain narrowband methods. Let the input one-dimensional series *x* be of length *N*. The SSA pipeline consists of four steps: embedding, singular value decomposition, diagonal averaging, and grouping reconstruction. The series *x* is first embedded into a trajectory matrix:

(13) $$X_{i,j}=x_{i+j-1},\;i=1,\ldots ,L,\;j=1,\ldots ,K$$

where *L* is the window length, *K* = *N* − *L* + 1, and *X* is the *L* × *K* Hankel matrix.

A truncated singular value decomposition is then performed on *X*, retaining the top *R* singular components:

(14) $$X\approx \sum_{r=1}^{R}\sigma_{r}U_{r}V_{r}^{T}$$

where *σ_r_* are the singular values arranged in descending order, and *U_r_* and *V_r_* are the corresponding left and right singular vectors. Each term *σ_r_U_r_V_r_^T^* forms a rank-one matrix of size *L* × *K*, which is restored to a one-dimensional series of length *N* through anti-diagonal averaging:

(15) $$x_{k}^{\left(r\right)}=\frac{1}{\left|D_{k}\right|}\sum_{(i,j)\in D_{k}}\sigma_{r}U_{r,i}V_{r,j}$$

where *D_k_* = {(*i*, *j*): *i* + *j* = *k* + 1} is the anti-diagonal index set. The *R* principal components are evenly divided into *n* groups in descending order of singular values, and the sub-modes are obtained by summing the components within each group. The SSA input is first z-score normalized to decouple scale sensitivity, and the original scale is restored after decomposition.

In this section, the power series in *P*_clean_ is decomposed by ICEEMDAN into 11 multi-scale components. K-means clustering is then performed on the joint indicators of permutation entropy and sample entropy, partitioning the components into high-, medium-, and low-frequency groups, with pure-noise modes softly suppressed via entropy-weighted reconstruction. SSA further refines the high-frequency group into four sub-modes, while the medium- and low-frequency groups are each reconstructed into a single component. The final output consists of six multi-scale sub-signals, which are concatenated along the channel dimension with the 8-dimensional exogenous features from EW-MPFI to form the 14-dimensional input to the DPC-Former backbone.

#### 3.4.4. Partition-Wise Fitting of the Decomposition

All three decomposition stages are applied independently to the training, validation, and test partitions using identical hyperparameters. No intrinsic mode function, cluster center, or singular component is estimated jointly across a partition boundary. However, ICEEMDAN and SSA are batch-mode and non-causal methods. Therefore, within each partition, the sub-signal at an earlier timestamp may depend on observations occurring later in the same partition. The reported results should accordingly be interpreted as retrospective offline forecasting performance rather than strictly causal real-time forecasting performance. The sensitivity of this remaining within-partition dependence to the length of the test-block decomposition window is quantified through the diagnostic reported in Section 4.6.8.

### 3.5. DPC-Former Backbone

#### 3.5.1. Optuna TPE Hyperparameter Optimization

Let the hyperparameter space be Θ. The Tree-structured Parzen Estimator (TPE) is employed to automatically search for the hyperparameters that minimize the quantile loss on the validation set. TPE partitions the observed points into a superior group and an inferior group according to their objective values, fits the density functions *l*(*θ*) and *g*(*θ*) respectively, and the next candidate point is determined by the following expression:

(16) $$\theta_{t+1}=\underset{\theta \in \Theta }{\arg\max}\frac{l(\theta )}{g(\theta )}$$

where *l*(*θ*) is the density of the superior group, and *g*(*θ*) is the density of the inferior group. The space Θ covers the learning rate, weight decay, dropout rate, stability loss weight, and network capacity parameters. The random seed is fixed throughout the search to ensure reproducibility.

#### 3.5.2. Decomposition-Aware Feature Refinement

The sub-modes and the EW-MPFI candidates are complementary, and their concatenation yields the extended feature set:

(17) $$F_{\mathrm{ext}}=F_{\mathrm{EW}}\cup {\left\{{\mathrm{HF}}_{k}\right\}}_{k=1}^{3}\cup \{\mathrm{MF},\mathrm{LF}\}$$

where $F_{\mathrm{EW}}$ denotes the EW-MPFI candidates, HF*_k_* is the *k*-th high-frequency sub-band, and MF and LF are the medium- and low-frequency components. The set $F_{\mathrm{ext}}$ is then evaluated through entropy-weighted fusion of three perspectives—mutual information, Tree-SHAP, and permutation importance—to quantify the marginal contribution of each feature to future power.

#### 3.5.3. DPC-Former Network Architecture

Let the exogenous input series be $x\in {\mathbb{R}}^{L\times D}$, where *L* is the look-back length, and *D* is the dimensionality of exogenous variables. This section models the data simultaneously along four dimensions: time, variable, scale, and operating regime. Channel-independent patch embedding is applied along the temporal dimension to extract temporal representations $h^{t}\in {\mathbb{R}}^{N\times D\times d_{h}}$, where *N* is the number of patches and *d_h_* is the hidden dimension. The channel dimension is then treated as the token axis, and self-attention is imposed along the variable dimension to establish the dependency between meteorological variables and wind power:

(18) $$(Q,K,V)=h^{t}W_{Q,K,V},\;h^{v}=\mathrm{softmax}\left(\frac{QK^{⊤}}{\sqrt{d_{h}}}\right)V$$

where W*_Q_*_,*K*,*V*_ are the learnable linear projection matrices. Sliding mean filters at 15 min, 1 h, and 4 h are then applied to the raw input *x* to extract the multi-scale meteorological representations m*_s_*. A stability classifier takes the surface-level temperature gradient, relative humidity, and diurnal phase as inputs and outputs a soft probability *g* ∈ Δ*^K^* that modulates the mixing weights of *K* experts:

(19) $$h^{\mathrm{moe}}=\sum_{k=1}^{K}g_{k}\cdot E_{k}\left(h^{v}⊕m_{s}\right)$$

where ⊕ denotes concatenation along the feature dimension, *E_k_* is the *k*-th expert network, and *g_k_* is the corresponding mixing weight. The classifier is simultaneously regularized by a weak-supervision cross-entropy constraint based on meteorological-state priors, with the weight λ_stab_.

For each horizon *h* and nominal quantile *τ*, the prediction head outputs the anchored residual-form quantile:

(20) $${\hat{q}}_{\tau }^{\;h}=y_{\mathrm{anchor}}+{\mathrm{Linear}}_{\tau }^{\;h}\left(h^{\mathrm{moe}}\right)$$

where *y*_anchor_ is the observation at the current time step, used to stabilize the residual magnitude. Under curtailment conditions, the standard pinball loss would drive the predictions downward. The curtailment mask *c*(*t*) ∈ {0, 1} is therefore introduced to re-weight the loss branches:

(21) $$L_{\mathrm{asym}}=(1-c)L_{\mathrm{pin}}(\tau ,r)+c\left[\max(0,r)+\gamma \max(0,-r)\right],\;r=y-{\hat{q}}_{\tau }$$

where *c*(*t*) = 1 indicates a curtailment time step, and *γ* ∈ (0, 1) is the attenuation coefficient for positive prediction bias during curtailment. The mask is evaluated at the target timestamp of each horizon, so that the weight applied to the prediction for step *t* + *h* is determined by *c*(*t* + *h*). It is therefore a label-side quantity, entering only the computation of the training loss, and it is not presented to the network at any point. In inference, the model produces the predictive quantiles from the input window alone, without curtailment information. The nominal significance level *α* is set to 0.20 for the 80% interval. The total loss is a linear combination of the multi-horizon pinball loss and the stability weak-supervision cross-entropy. AdamW is adopted for training, with weight decay disabled for bias and LayerNorm parameters.

This section concatenates the 8-dimensional exogenous features and the 6-dimensional sub-signals along the channel dimension to form a 14-dimensional input. The backbone consists of three components: cross-variable attention (CVA), multi-scale parallel encoding (MNE), and the stability-gated mixture-of-experts (StabGate). Four core hyperparameters, namely the learning rate, weight decay, dropout rate, and stability supervision weight, are automatically searched by Optuna TPE within a budget of 50 trials. The network is jointly trained at three horizons of 15 min, 1 h, and 4 h using a horizon-weighted pinball loss, with the three horizons produced in a single forward pass. The network is independently run with 50 random seeds to characterize training stochasticity, with all seeds sharing the same network architecture and hyperparameters while differing in initialization and data shuffling.

### 3.6. NGBoost Residual Probabilistic Layer

DPC-Former outputs only three nominal quantiles at each horizon, which is insufficient to characterize the complete predictive distribution. NGBoost imposes natural gradients under the Fisher information metric, enabling the mean and dispersion parameters to be updated on a consistent scale [42]. This study builds a residual probabilistic layer based on NGBoost on top of the three-quantile output of DPC-Former, with the Gaussian family chosen as the predictive distribution. The natural gradient update of the negative log-likelihood is defined as follows:

(22) $$\tilde{\theta }\leftarrow \theta -\eta \cdot I_{F}{\left(\theta \right)}^{-1}\nabla_{\theta }L(\theta ;y)$$

where $I_{F}(\theta )=E_{y}[-\nabla_{\theta }^{2}\log p(y|\theta )]$ is the Fisher information matrix, and *η* is the line-search step size. An independent NGBoost model is trained for each horizon *h*, with CART regression trees as the base learners, the Gaussian family as the predictive distribution, the three quantiles output by DPC-Former as the input, and the actual power as the target:

(23) $$\left({\hat{\mu }}^{\;h},{\hat{\sigma }}^{\;h}\right)={\mathrm{NGB}}^{\;h}\left({\hat{q}}_{0.1}^{\;h},{\hat{q}}_{0.5}^{\;h},{\hat{q}}_{0.9}^{\;h}\right)$$

Training adopts the continuous ranked probability score (NCRPS) as the early-stopping criterion. The prediction interval at the 1 − *α* confidence level is derived from the Gaussian quantile function, with *z*_1−*α*/2_ being the 1 − *α*/2 quantile of the standard normal distribution.

In this section, the concatenation of [q_10_, q_50_, q_90_] and the exogenous features serves as the input to an independent NGBoost model trained for each horizon, with the parameters of the Gaussian distribution learned through natural gradients. The predictive distributions from 50 random seeds are then averaged in an ensemble to produce the final probabilistic output.

### 3.7. Evaluation Metrics

Six metrics are adopted in this study to evaluate the models. The normalized mean absolute error (NMAE) and the normalized root mean squared error (NRMSE) reflect point-forecasting accuracy. The normalized continuous ranked probability score (NCRPS) provides a comprehensive characterization of probabilistic-forecasting accuracy. The prediction interval coverage probability (PICP) and the normalized prediction interval average width (PINAW) jointly assess interval calibration and sharpness. The probability integral transform (PIT) examines the consistency of distributional shape. All dimensional metrics are normalized by the installed capacity *C*_cap_.

NMAE and NRMSE are defined as:

(24) $$\mathrm{NMAE}=\frac{1}{M}\sum_{i=1}^{M}\frac{\left|y_{i}-{\hat{\mu }}_{i}\right|}{C_{\mathrm{cap}}}\times 100\%$$

(25) $$\mathrm{NRMSE}=\frac{1}{C_{\mathrm{cap}}}\sqrt{\frac{1}{M}\sum_{i=1}^{M}{\left(y_{i}-{\hat{\mu }}_{i}\right)}^{2}}\times 100\%$$

Under the Gaussian distribution, CRPS admits a closed-form expression. Its normalized form, NCRPS, is defined as CRPS divided by *C*_cap_:

(26) $$\mathrm{NCRPS}=\frac{\sigma }{C_{\mathrm{cap}}}\left[z\left(2\Phi (z)-1\right)+2\varphi (z)-\frac{1}{\sqrt{\pi }}\right]\times 100\%,\;z=\frac{y-\mu }{\sigma }$$

PICP and PINAW at a nominal confidence level 1 − *α* are defined as:

(27) $${\mathrm{PICP}}_{1-\alpha }=\frac{1}{M}\sum_{i=1}^{M}I\left\{y_{i}\in \left[{\hat{\mu }}_{i}\pm z_{1-\alpha /2}{\hat{\sigma }}_{i}\right]\right\}\times 100\%$$

(28) $${\mathrm{PINAW}}_{1-\alpha }=\frac{2z_{1-\alpha /2}}{MC_{\mathrm{cap}}}\sum_{i=1}^{M}{\hat{\sigma }}_{i}\times 100\%$$

The pinball loss is used to evaluate the overall accuracy of quantile forecasts. Its normalized form is defined as:

(29) $$\mathrm{Pinball}=\frac{1}{MC_{\mathrm{cap}}}\sum_{i=1}^{M}\rho_{\tau }\left(y_{i}-{\hat{q}}_{\tau ,i}\right)\times 100\%,\;\rho_{\tau }(u)=u\left(\tau -I\{u<0\}\right)$$

PIT is defined as:

(30) $${\mathrm{PIT}}_{i}=\Phi \left(\frac{y_{i}-{\hat{\mu }}_{i}}{{\hat{\sigma }}_{i}}\right)$$

where *M* is the number of test samples, *y_i_* is the ground-truth value, ${\hat{\mu }}_{i}$ and ${\hat{\sigma }}_{i}$ are the mean and standard deviation output by NGBoost, ${\hat{q}}_{\tau ,i}$ is the quantile prediction at level *τ*, Φ and *φ* denote the cumulative distribution function and the probability density function of the standard normal distribution, respectively, and I{·} is the indicator function. 

## 4. Experiments and Results

### 4.1. Dataset and Experimental Setup

#### 4.1.1. Data Source

The data used in this study were collected from a 130 MW mountainous wind farm in Sichuan, China, and provided under a collaboration agreement with the wind farm operator. The turbines have a hub height of 110 m, with cut-in, rated, and cut-out wind speeds of 3 m/s, 15 m/s, and 22 m/s, respectively. The dataset spans 91 days from 1 March to 31 May 2025 at a 15-min resolution, producing 96 observations per day and 8736 time steps in total. Wind speed is recorded synchronously at two heights of 10 m and 100 m, with the 100 m measurement height being close to the hub height and thus directly representative of the inflow at the rotor. The meteorological variables used as exogenous inputs in this study are observations recorded on-site at the wind farm. During forecasting, the model takes the historical endogenous power series and the concurrently observed meteorological variables as inputs. The meteorological variables include wind speed, wind direction, temperature, relative humidity, and atmospheric pressure, and the complete variable list is provided in Table 1.

#### 4.1.2. Data Preprocessing

**Figure 2:**
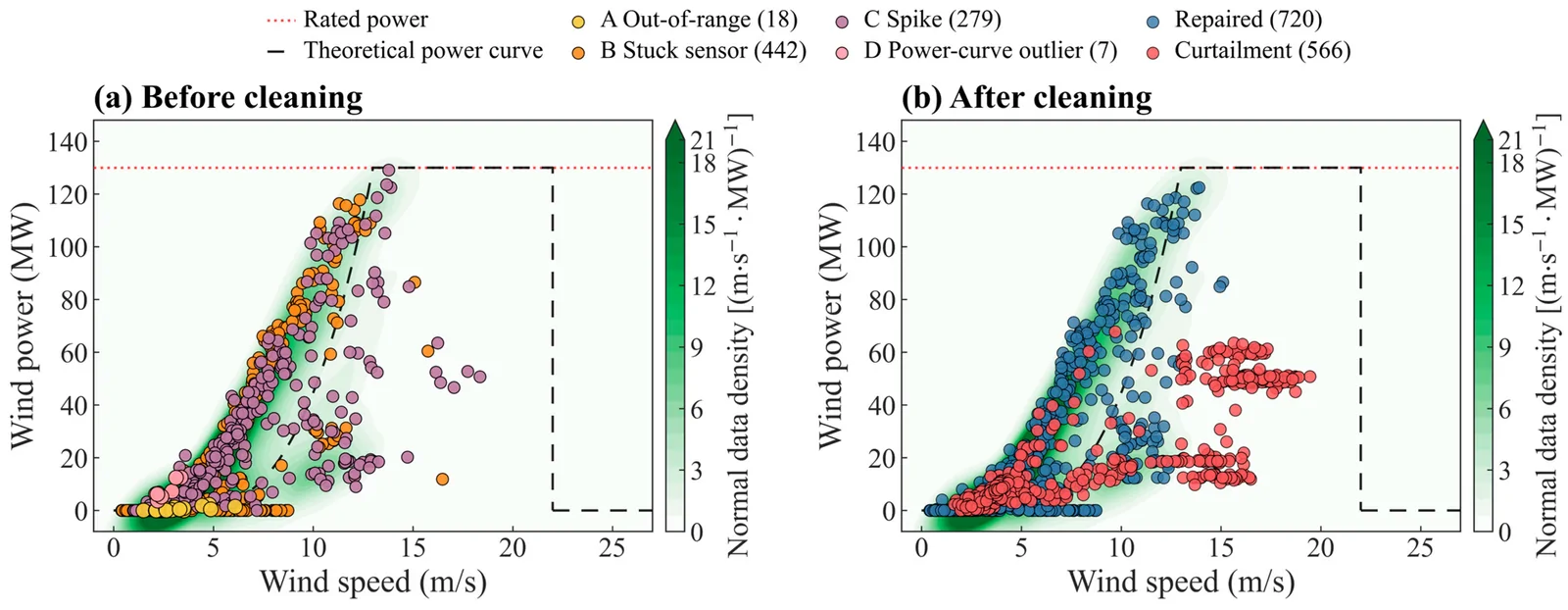
Wind speed versus power scatter of the raw and cleaned datasets. (**a**) Before cleaning, there are four categories of anomalies flagged. (**b**) After cleaning, the repaired and curtailment samples were highlighted.

The raw data contains anomalies and missing values caused by sensor failures and communication packet loss. A two-stage strategy is adopted to perform rule-based detection and hierarchical imputation, with the results shown in Figure 2. In the detection stage, physical criteria are established based on the cut-in speed of 3 m/s, the rated speed of 15 m/s, the cut-out speed of 22 m/s, and the installed capacity of 130 MW. The four rules A through D identify 18, 442, 279, and 7 anomalous samples, respectively, totaling 746 samples and accounting for 8.54% of the entire dataset. These anomalies are mainly distributed along the upper and lower edges of the main band of the power curve. In the imputation stage, the flagged samples are repaired in tiers according to the gap length, and the repaired samples revert to the vicinity of the theoretical power curve, with the overall distribution conforming to the operating characteristics of the turbines.

Curtailment samples are different in nature from the anomalies described above: they represent the actual output of the turbines and deviate from the potential generation only because of dispatch restrictions, with the meteorological information remaining valid. They are therefore preserved without modification during preprocessing, yielding 566 samples and accounting for 6.48% of the dataset.

### 4.2. Feature Selection Results

**Figure 3:**
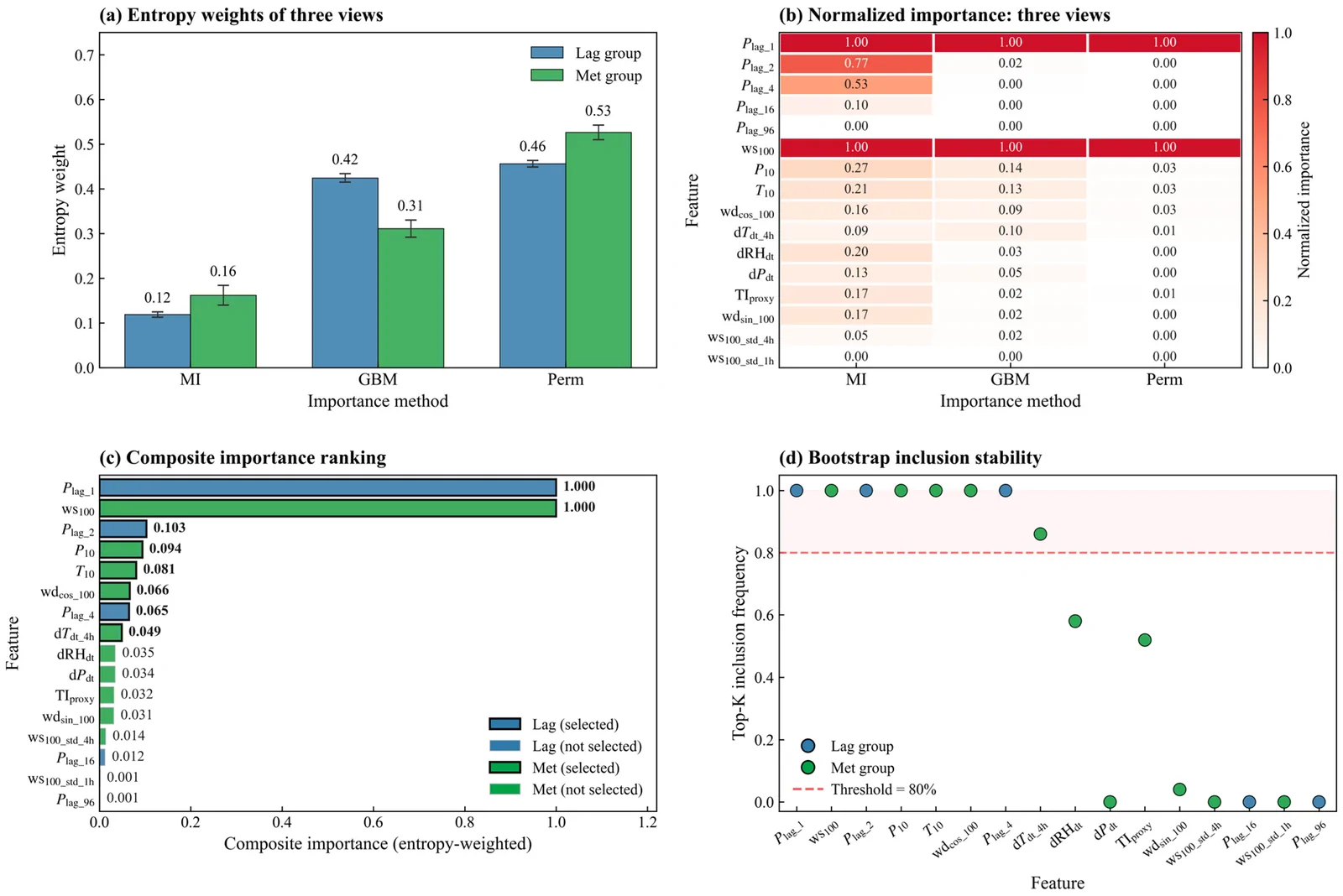
EW-MPFI three-perspective fusion and stability screening results. (**a**) Entropy weights of the three views. (**b**) Normalized importance matrix across the three views. (**c**) Composite importance ranking. (**d**) Bootstrap inclusion stability.

The exogenous inputs for wind power forecasting encompass multi-source meteorological and historical power variables, with the candidate dimensionality being high and the predictive value varying markedly across variables. Through feature engineering involving lagging, differencing, and trigonometric transformations, the original 23-dimensional meteorological and power observations are processed into a 16-dimensional candidate feature set, on which EW-MPFI performs the final selection. The results are presented in Figure 3.

In the entropy-weighted fusion stage, the permutation importance perspective exhibits the highest discrimination in both groups, as shown in Figure 3a, with w_Perm reaching 0.46 in the lag group and 0.53 in the meteorological group. Mutual information receives the lowest weight, as it tends to assign similarly high scores to multiple features within the saturation region. Across 50 Bootstrap resamples, the standard deviations of the three-perspective weights remain below 0.025, confirming the stability of the estimates. The normalized score matrix is shown in Figure 3b, where *P*_lag_1_ and ws_100_ dominate across all three perspectives in the two groups, respectively, reflecting the governing roles of short-term autocorrelation and hub-level wind speed. The composite ranking and the stability test are presented in Figure 3c,d. After applying the joint truncation of the 90% cumulative contribution and r ≥ 0.8, eight features are retained from the 16 candidates: *P*_lag_1_, *P*_lag_2_, *P*_lag_4_, ws_100_, *P*_10_, *T*_10_, wd_cos_100_, and d*T*_dt_4h_. The selected subset covers short-memory autocorrelation, the dominant driver of hub-level wind speed, the cosine projection of wind direction, and the four-hour temperature rate of change, consistent with the physical understanding that short-term wind power is jointly governed by autocorrelation, wind speed energy, and thermal stability.

### 4.3. Signal Decomposition Results

The wind power series exhibits multi-scale superposition characteristics, comprising high-frequency turbulent disturbances, mid-frequency intraday structures, and low-frequency trend components. A single model struggles to accommodate all scales. This section, therefore, adopts a three-stage strategy of ICEEMDAN primary decomposition, multivariate entropy-weighted reconstruction, and SSA secondary decomposition to decompose the series into multi-scale sub-components, alleviating the inductive bias conflicts across scales.

Within the training partition, *P*_clean_ is decomposed by ICEEMDAN into 10 IMFs and one residual, with the ensemble size set to 100, the white noise standard deviation set to 0.2, and the maximum number of modes set to 20. The validation and test partitions are decomposed independently using the same hyperparameters. The normalized permutation entropy (PE) and sample entropy (SE) of each component are listed in Table 2. K-means clustering with *K* = 3 is then applied to the two-dimensional PE–SE features. Sorting the cluster centers in descending order of PE yields the high-frequency group HF (IMF1, IMF2), the medium-frequency group MF (IMF3 through IMF6), and the low-frequency group LF (IMF7 through IMF10 and the residual).

After z-score normalization, the HF group is fed into SSA with a window length *L* = 60. The top 24 singular components are retained and evenly divided into three subgroups in descending order, yielding the sub-modes HF1, HF2, and HF3, with standard deviations of 3.67, 2.15, and 1.11 MW, respectively. The SSA residual has a standard deviation of 0.56 MW, accounting for 11.4% of the input standard deviation, and is preserved as an independent component HF_RES_.

**Figure 4:**
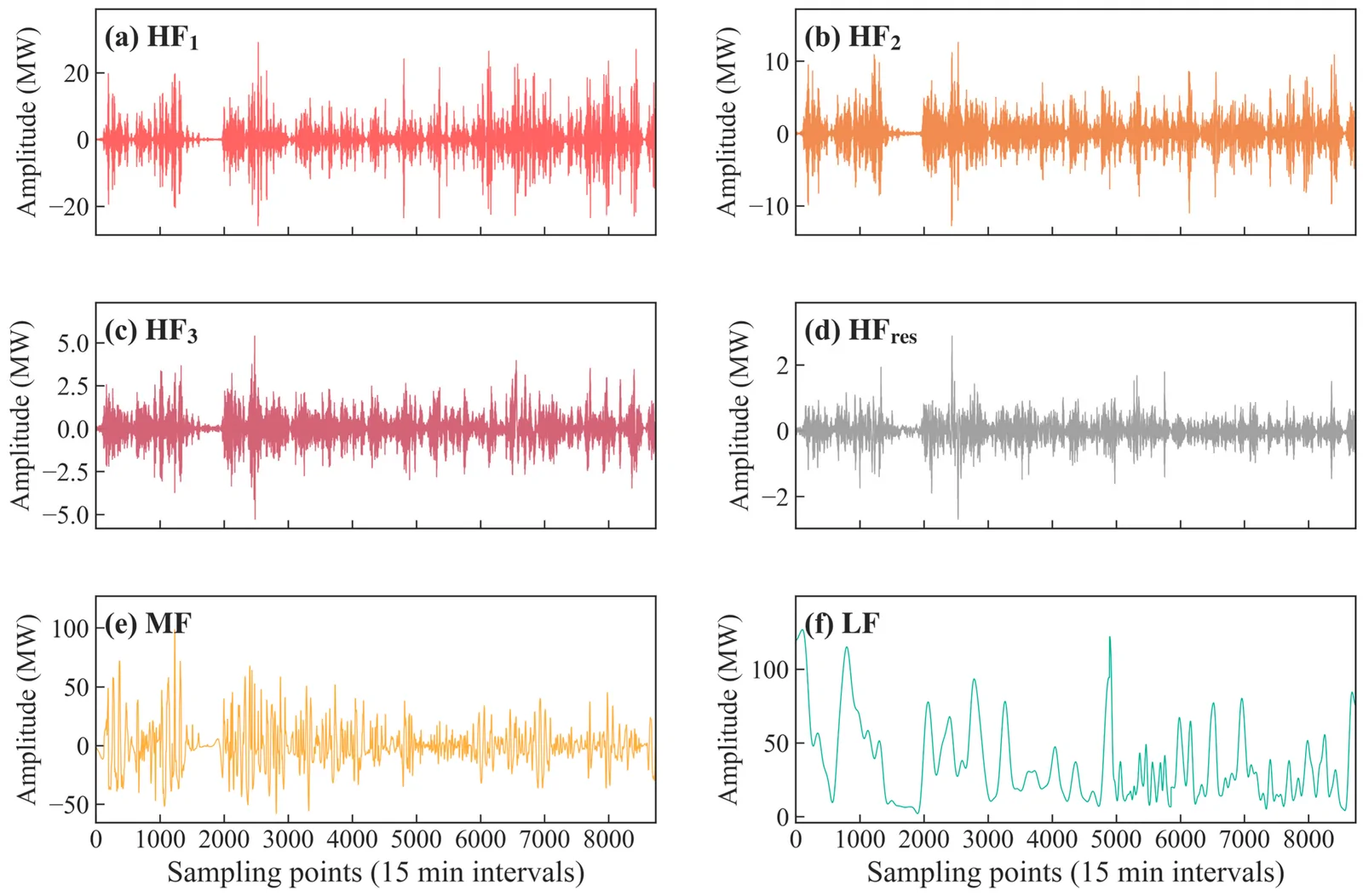
Multi-scale sub-signal set after the three-stage decomposition. (**a**) HF_1_. (**b**) HF_2_. (**c**) HF_3_. (**d**) HF residual. (**e**) MF. (**f**) LF.

After the three-stage processing, *P*_clean_ is reorganized into six sub-components: HF_1_, HF_2_, HF_3_, HF_RES_, MF, and LF. The final multi-scale sub-signal set is presented in Figure 4 and, together with the selected exogenous features, serves as the input to the forecasting model, with the actual power *P*_actual_ as the prediction target.

### 4.4. Backbone Forecasting Performance

DPC-Former is fitted on the training set, with the validation set used solely for early stopping. The three horizons H ∈ {1, 4, 16} steps and the three quantiles *τ* ∈ {0.10, 0.50, 0.90} share the same backbone. The loss is the sum of the multi-horizon pinball losses, weighted by the monotonically decreasing coefficients {2.0, 1.5, 1.0} to compensate for the gradient dominance of longer horizons. AdamW is adopted as the optimizer, with weight decay disabled for bias and LayerNorm parameters. The core hyperparameters are jointly searched by Optuna TPE within a budget of 50 trials, with the learning rate space restricted to [10^−5^, 10^−3^] to avoid the under-convergence instability in the low-learning-rate region. The complete training protocol is provided in Table 3.

**Figure 5:**
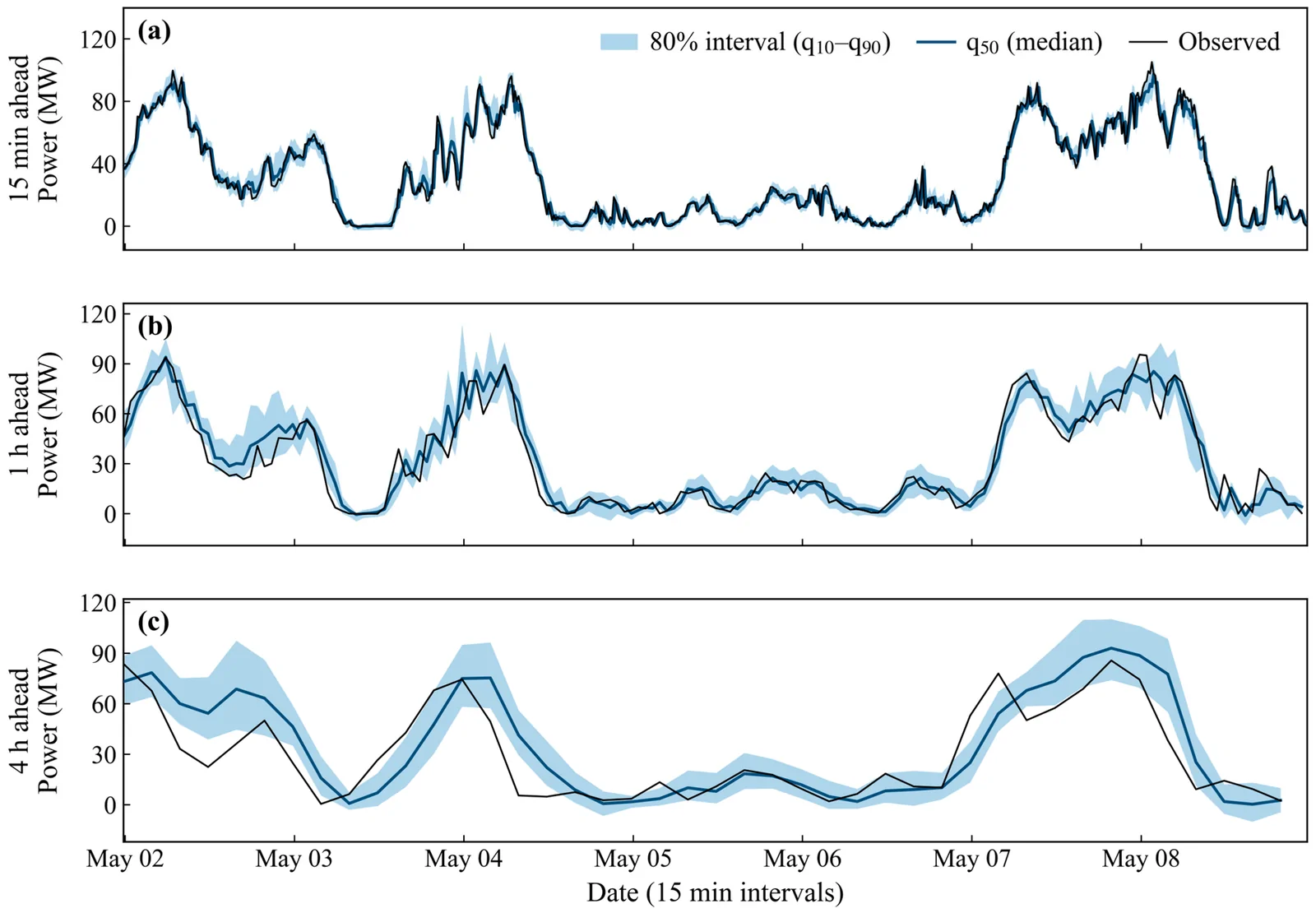
Horizon-wise probabilistic forecasting visualization over a representative test window. (**a**) 15 min ahead. (**b**) 1 h ahead. (**c**) 4 h ahead.

The eight-dimensional exogenous features selected by EW-MPFI and the six-dimensional sub-signals output by the three-stage decomposition are concatenated along the channel dimension to form a 14-dimensional input, with the aggregate power serving as the single supervised target. After training, the input importance on the test set is measured through entropy-weighted fusion of three perspectives, namely KSG mutual information, Tree-SHAP, and elastic-net permutation importance. The top ten features in the composite ranking include three decomposition sub-signals—LF, MF, and HF1—confirming that the model substantively utilizes the decomposed components. On the test set, the aggregate performance reaches an NMAE of 5.21%, an NRMSE of 7.38%, and a pinball loss of 1.69%, with the 80% coverage directly constructed from q_10_ and q_90_ being 0.77, and the average interval width accounting for 15.52% of the installed capacity. The horizon-wise performance is provided in Table 4, and the probabilistic forecasts of a representative window are shown in Figure 5.

### 4.5. Probabilistic Forecasting Performance

To obtain a complete predictive distribution and support closed-form strict NCRPS evaluation, this study builds a two-stage NGBoost residual probabilistic layer on top of the three-quantile prediction of DPC-Former. In the first stage, the DPC-Former outputs are frozen. In the second stage, the concatenation of [q_10_, q_50_, q_90_] and the exogenous features is used as the input, with an independent NGBoost trained for each forecasting horizon. The Gaussian distribution *N*(*μ*, *σ*^2^) is adopted as the probabilistic family, and the negative log-likelihood is optimized through natural gradients. Each NGBoost model employs 500 decision trees, a learning rate of 0.01, and an early-stop patience of 30 rounds, with one independent model trained for each of the three horizons.

**Figure 6:**
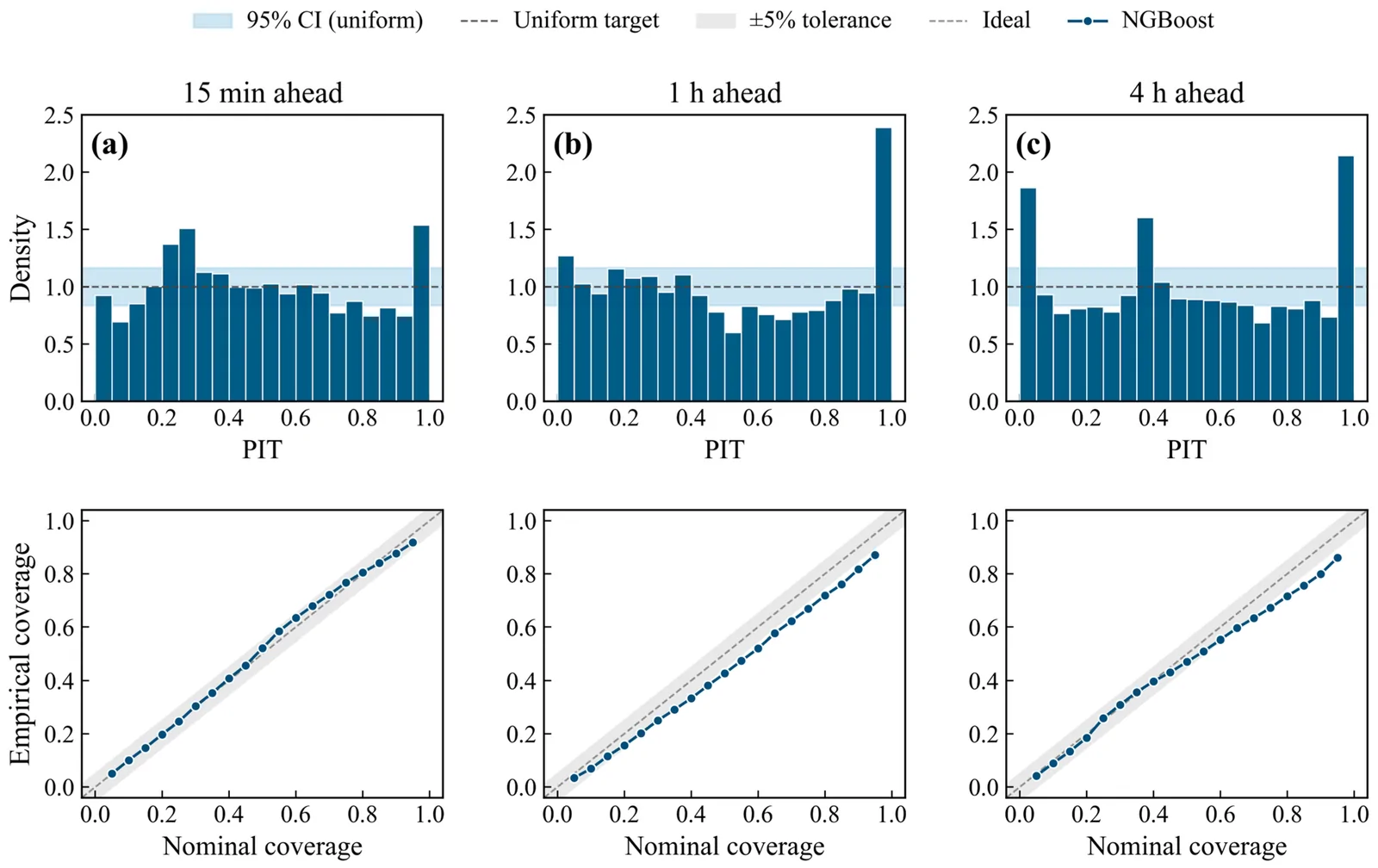
Calibration diagnostics of the NGBoost probabilistic layer, showing PIT histograms (**top**) and reliability diagrams (**bottom**) for three forecasting horizons. (**a**) 15 min ahead. (**b**) 1 h ahead. (**c**) 4 h ahead.

The closed-form NCRPS on the test set is 3.74%. The NGBoost residual probabilistic layer preserves the point-forecasting accuracy while producing a complete conditional probability distribution. Under the nominal confidence levels of 80% and 90%, the empirical coverage probabilities reach 0.84 and 0.93, respectively. The mild over-coverage reflects the compounding effect of the seed-wise variance and the conditional variance output by NGBoost, which widens the interval beyond the nominal value. The standardized residual standard deviation *z* = 0.95 is close to the optimal Gaussian value of 1.0, and the reliability curve deviates from the diagonal by no more than 0.10 across all horizons. The PIT histogram is overall close to uniform, with only a slight right skew at the 4 h horizon, reflecting the mildly positive-skewed non-Gaussian characteristics of the residual distribution during long-horizon power ramp events. The deviation, however, is not substantial. The horizon-wise probabilistic metrics are reported in Table 5, and the probabilistic calibration diagnostics are shown in Figure 6.

### 4.6. Comparative Experiments and Ablation Analysis

#### 4.6.1. Ablation on Data Preprocessing, Feature Engineering, and Signal Decomposition

The input processing of DPC-Former consists of three core modules: anomaly preprocessing, EW-MPFI feature selection, and three-stage signal decomposition. This section designs 10 end-to-end variants for ablation comparison. All variants are independently trained with 50 random seeds and then ensemble-averaged. The results are reported in Table 6.

The full method I-P-S achieves the best performance. Along the three-stage strategy, simplifying step by step, the NMAE rises by 6.33% after removing the SSA secondary decomposition, and by a cumulative 10.94% after further removing the multivariate entropy-weighted reconstruction and retaining only the ICEEMDAN primary decomposition. In horizontal comparison against other single-stage decomposition methods, the three-stage strategy outperforms SSA, CEEMDAN, VMD, and WPD by 12% to 16%.

At the module level, completely removing the signal decomposition, the anomaly preprocessing, and the EW-MPFI feature selection raises the NMAE by 25.36%, 19.96%, and 24.57%, respectively. The three degradations fall within the same order of magnitude.

#### 4.6.2. Model Comparison

**Figure 7:**
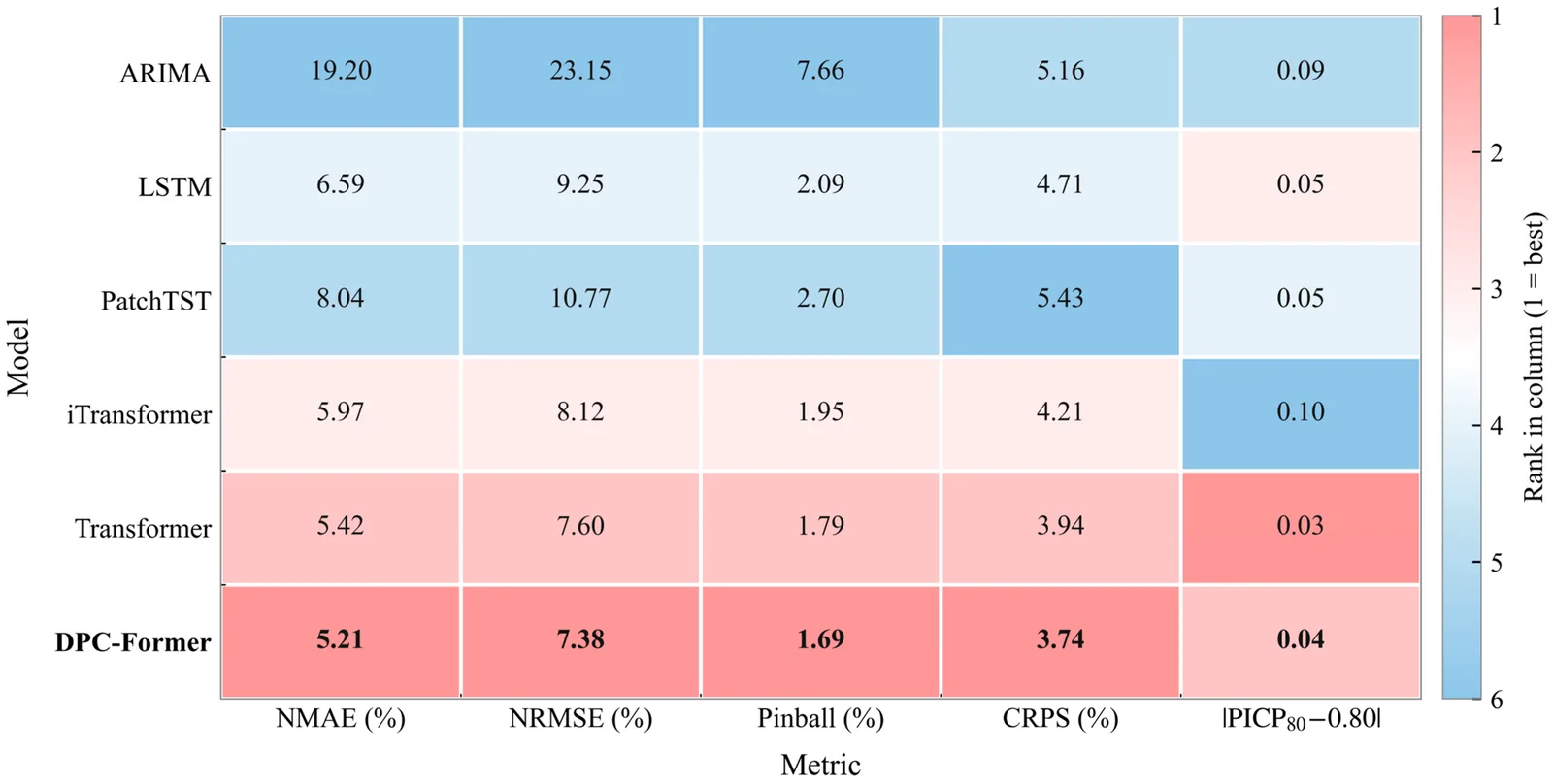
Heatmap comparison of baseline models. Bold values highlight the performance of the proposed DPC-Former model.

DPC-Former is compared against four deep-learning baselines—LSTM, Transformer, iTransformer, and PatchTST—and one statistical baseline, ARIMA. The baselines are implemented following the original configurations of their source publications, with the input features kept consistent with those of DPC-Former. All models are independently trained with 50 random seeds and then ensemble-averaged. The parameter counts of the five deep models range from 0.21 M to 0.59 M, all within the same order of magnitude. The results are reported in Table 7, and the five-dimensional metric comparison is shown in Figure 7.

DPC-Former achieves the best performance on all four core metrics: NMAE, NRMSE, pinball loss, and NCRPS. On the calibration metric |PICP_80_-0.80|, DPC-Former is essentially on par with Transformer, and both significantly outperform iTransformer. DPC-Former is the only model among the six that simultaneously ranks within the top two on both point-forecasting accuracy and probabilistic calibration.

A grouped comparison by baseline type reveals further insights. The second-best baseline, Transformer, is the closest to DPC-Former overall, confirming the effectiveness of the basic Transformer architecture for wind power forecasting. iTransformer and PatchTST represent two improvement directions, namely variable tokenization and channel independence, respectively, yet neither outperforms the standard Transformer on this task. LSTM, constrained by the limited long-sequence modeling capacity of its recurrent structure, exhibits an NCRPS 25.94% higher than that of DPC-Former. The statistical baseline ARIMA shows NMAE and NRMSE values approximately three to four times those of the best deep-learning method, confirming the necessity of nonlinear modeling for the wind-speed-to-power mapping.

#### 4.6.3. Multi-Seed Robustness

To assess the stability of the DPC-Former framework with respect to random initialization, the complete training–evaluation pipeline of DPC-Former and NGBoost is repeated with 50 independent random seeds. The statistical results are summarized in Table 8.

Across the 50 runs, the 95% confidence interval width on each of the three core metrics (NCRPS, NMAE, and NRMSE) remains below 5% of the corresponding mean. The mean PICP_80_ of single-seed DPC-Former + NGBoost instances is 0.79, closely matching the nominal level of 0.80. These results indicate that the framework is highly insensitive to random initialization and delivers robust performance. Ensemble-averaging the predictive outputs from the 50 seeds yields the main results, which further improve NCRPS, NMAE, and NRMSE by 7.40%, 7.00%, and 13.70%, respectively, compared with the single-seed mean. This reflects the dual benefit of ensembling in both variance reduction and centralized estimation. Notably, the ensemble PICP_80_ rises from 0.79 to 0.84. While preserving the aleatoric uncertainty captured by each seed, the ensemble additionally incorporates the seed-wise variance as epistemic uncertainty, widening the final prediction interval. This is the expected behavior of ensembling and, in engineering practice, translates into a more conservative interval width.

#### 4.6.4. Probabilistic Layer Component Comparison

To verify the design advantages of NGBoost, this section fixes the point-forecasting source as the 50-seed ensemble output of DPC-Former and replaces only the conditional distribution modeling approach. Six variants V1 through V6 are designed, covering NGBoost, zero-cost Gaussian, quantile regression, kernel density estimation, Bayesian linear regression, and MC Dropout. The configurations are listed in Table 9. In the table, *z*_std_ denotes the standardized residual standard deviation, with an ideal value of 1.0.

V1, the method proposed in this study, achieves the best performance on all four core metrics: NMAE, NRMSE, pinball loss, and NCRPS, with an improvement of 1.5% to 2.4% over the second-best variant V2. V2 shows aggregate metrics close to V1, but its *z*_std_ of 1.14 deviates from the ideal value, reflecting that the *σ* obtained by linear interpolation of quantiles is overly optimistic. V1, by explicitly modeling the residual variance through NGBoost, keeps *z*_std_ closely matched with the ideal value at 0.95.

The remaining four variants each exhibit notable shortcomings. V3, unable to access deep-layer features, suffers from a severely over-optimistic *z*_std_. V4, under the homoscedasticity assumption, fails to capture state-dependent characteristics. V5, performing only a linear mapping, leads to a substantial degradation in NMAE. V6 exhibits under-coverage of PICP_80_ at the 4 h horizon. V1 is therefore adopted as the final probabilistic output layer in this study.

#### 4.6.5. Internal Component Ablation

To quantify the marginal contribution of each internal component of DPC-Former, five variants are designed by removing one component at a time from the full method M1, forming a single-component ablation. All variants are independently trained with 50 random seeds and then ensemble-evaluated. M1 reuses the results from Section 4.6.2. The results are reported in Table 10.

The components, ranked by marginal contribution in descending order, are CVA, MNE, StabGate, and Optuna. Removing CVA has the largest impact, raising the NMAE by 4.61%. Removing MNE comes next, raising the NMAE by 3.45%. The marginal contributions of StabGate and Optuna are smaller in magnitude but consistently positive.

Even the worst-performing variant M4 attains an NMAE of 5.45%, which remains essentially on par with the best baseline Transformer in Section 4.6.2 and outperforms the remaining baselines. This indicates that the accuracy advantage of DPC-Former originates from the overall architectural design rather than from any single component.

#### 4.6.6. Computational Cost

To evaluate the engineering deployment feasibility of DPC-Former, this section compares the parameter count, training time, and inference latency of each model on an NVIDIA RTX 5060 GPU. The results are reported in Table 11.

DPC-Former has 0.34 M parameters, which is only 58% of that of PatchTST, yet delivers higher accuracy, confirming that the performance gain originates from the architectural design rather than from capacity expansion. Even when including the two additional steps of the NGBoost probabilistic layer and the 50-seed ensemble, the total modeling time remains below 50 min, which is still faster than the approximately 88-min training cost of PatchTST under the same seed scale. In conclusion, the single-sample latency is 0.039 ms, on the same sub-millisecond order as the other deep models, far below the requirement of real-time dispatch at the 15-min resolution. In summary, DPC-Former achieves leading accuracy without incurring substantial computational cost.

#### 4.6.7. Cross-Partition Decomposition-Scope Control

ICEEMDAN and SSA are batch-mode transforms. An implementation that fitted them over the full record would therefore allow each sub-signal to be influenced by observations occurring after its own timestamp, and the resulting sub-signals would carry information unavailable at the forecast origin. The decomposition in this work is fitted independently within each chronological partition, as described in Section 3.4.4, so no such transfer occurs between the training, validation, and test blocks. To quantify the effect of cross-partition decomposition fitting, the alternative implementation was deliberately constructed as a control.

In configuration L1, a single ICEEMDAN, a single entropy-based grouping, and a single SSA were applied to the entire 91-day record, with the training, validation, and test blocks decomposed together. Configuration L0 is the partition-wise implementation used throughout this work. The two are structurally distinct rather than a relabeling of one another: L1 resolves 11 intrinsic modes plus a residual, with high-, medium-, and low-frequency group sizes of 3, 3, and 6, whereas the training-partition decomposition reported in Table 2 resolves 10 intrinsic modes plus a residual with group sizes of 2, 4, and 5. The backbone was retrained from scratch under each configuration using five common random seeds, with all other settings held fixed. Because these are five individually trained models rather than a 50-seed ensemble, the values are not directly comparable with those of Table 6 and Table 7.

The results appear in Table 12. Configuration L1 is more accurate at every forecast horizon and on every metric. Test NMAE decreases from 2.689% to 1.950% at 15 min, from 5.263% to 2.723% at 1 h, and from 8.804% to 7.074% at 4 h, a horizon-averaged reduction of 1.670 percentage points, or 29.9% in relative terms. NRMSE and NCRPS behave identically, and the ordering holds in each of the five seeds at each of the three horizons.

Two conclusions follow. First, fitting the decomposition across the complete record confers a large and systematic accuracy advantage, confirming that the decomposition scope is material rather than nominal. Second, all principal results reported in this study use configuration L0 and therefore do not include this cross-partition advantage. This control, however, addresses only information transfer across the training, validation, and test boundaries; it does not eliminate the dependence of earlier sub-signal values on later observations within the test block. The remaining within-test-block sensitivity is therefore quantified separately in Section 4.6.8.

#### 4.6.8. Test-Block Decomposition-Window Diagnostic

Although the partition-wise protocol prevents the decomposition from being estimated jointly across the training, validation, and test boundaries, the batch decomposition of the complete test block remains non-causal within that block. To quantify the sensitivity associated with this remaining dependence, the complete 33-day test block is divided into sequential non-overlapping windows corresponding to the complete block, approximately 7 days, 3 days, and 1 day. Any trailing remainder is retained as the final shorter window, and each window is decomposed independently. The trained DPC-Former checkpoint with seed 42, the meteorological inputs, and all non-decomposition settings are held fixed, and only the six decomposition-derived test channels are regenerated. The ICEEMDAN and SSA settings are kept consistent with the submitted protocol. To preserve the semantic correspondence of the high-, medium-, and low-frequency channels presented to the pretrained model, the PE-SE cluster centers estimated from the training partition are frozen and reused for every test sub-window.

The diagnostic uses the backbone quantile outputs from one fixed checkpoint rather than the 50-seed NGBoost ensemble used for the headline results. Its absolute metric values are therefore not directly comparable with the NMAE of 5.21%, NCRPS of 3.74%, and PICP_80_ of 0.84 reported for the full framework. The purpose of the experiment is instead to measure the relative change caused by shortening the test-block decomposition window while holding the forecasting model fixed.

As shown in Table 13, the approximately weekly setting produces no detectable change in horizon-averaged NMAE after rounding, while the three-day setting increases NMAE by 0.150 percentage points. Both changes are smaller than the across-seed standard deviation of 0.248 percentage points observed in the five-seed L0 control. In contrast, the one-day setting increases NMAE by 0.951 percentage points and degrades the other accuracy and calibration metrics. This comparison is provided as a reference scale rather than as a paired significance test because the window diagnostic uses one fixed checkpoint.

The daily degradation should be interpreted as an upper bound on the possible effect of within-test-block look-ahead rather than as a direct estimate of leakage. Shortening the window not only reduces the later observations available to the batch decomposition but also weakens its ability to resolve longer-period structures, as indicated by the reduction in ICEEMDAN modes from 10 to 5–6. The observed degradation therefore combines reduced within-block look-ahead and reduced decomposition resolution. Overall, performance remains stable at approximately weekly and three-day cadences, whereas a daily cadence produces a material deterioration.

## 5. Discussion

This study addresses the problem of multi-horizon probabilistic forecasting for mountainous wind farms by constructing a forecasting framework that integrates three coordinated modules: EW-MPFI feature selection, three-stage signal decomposition, and the DPC-Former backbone together with the NGBoost probabilistic layer. Validated on field measurements, the proposed method consistently achieves the best results on four point-forecasting metrics and five probabilistic-forecasting metrics. The 50-seed ensemble strategy further delivers a stable 7–14% performance improvement over single-seed models. The following discussion examines the three dimensions of the framework in turn: the front-end processing, the backbone architecture, and the probabilistic layer, and states the boundary of each. The data are drawn from a single mountainous wind farm in Sichuan, China, comprising 91 days of observations from the spring of 2025, and this scope should be borne in mind throughout.

The ablation experiments reveal the source of contribution from each module. Removing the signal decomposition raises the NMAE by 25.36%, removing EW-MPFI raises it by 24.57%, while removing CVA and MNE only raise it by 4.61% and 3.45%, respectively. This indicates that the front-end data processing stages contribute more than 70% of the total performance gain, consistent with the strong non-stationarity and high-dimensional redundancy of mountainous wind power series. In signal decomposition, Sun et al. used sample entropy to select the single most unpredictable mode for secondary ICEEMDAN refinement [43], yet the remaining modes are passed forward unaltered, and entropy does not enter the recombination. Zhang et al. clustered the primary modes by four entropies into a complex and a simple group and refined the complex group by VMD [44], yet the grouping is binary, and its reconstruction is an unweighted sum.

In contrast, the three-stage strategy proposed in this study partitions the modes into three complexity bands, so that medium- and low-frequency structure remains separable at the backbone input; retains the permutation entropy values as scale-matched reconstruction weights rather than a uniform sum; and applies SSA rather than VMD, which removes the preset mode count that Zhang et al. identify as a limitation of their own method. In feature selection, Liao et al. found the most trustworthy attribution technique to be model-dependent, permutation importance ranking first for neural networks and SHAP for tree models [45], yet no selection rule was derived, leaving the ranking dependent on the method chosen. The EW-MPFI proposed in this study fuses KSG mutual information, Tree-SHAP, and elastic-net permutation importance through entropy-weighted aggregation across three principal complementary perspectives, augmented with Bootstrap stability validation, reducing the fitting burden of the backbone from the source. The front-end decomposition is implemented independently within each chronological partition, which prevents joint estimation across the training, validation, and test blocks but does not make the batch operators causal within a block. The diagnostic in Section 4.6.8 shows that performance remains stable at approximately weekly and three-day cadences, whereas a daily cadence produces a material degradation. Because shortening the window also reduces the number of resolvable ICEEMDAN modes, the daily degradation reflects the combined effects of reduced within-block look-ahead and reduced decomposition resolution rather than a pure estimate of information leakage. The reported metrics should therefore be interpreted as offline best-case results; strictly causal rolling- or expanding-window evaluation remains necessary before operational deployment.

The marginal contribution of the backbone architecture, though relatively limited, is structurally irreplaceable within the overall framework. Under complex mountainous terrain, meteorological variables such as wind speed, wind direction, and temperature exhibit strong cross-variable correlations under the joint influence of orographic lifting and diurnal thermal effects, and this cross-variable structural information constitutes a key factor of power fluctuations. Prior work in photovoltaic power forecasting has explicitly treated the variable dimension as attention tokens, verifying the capability of cross-variable self-attention for modeling multivariate dependencies in energy time series [46]. Building on this foundation, the CVA in this study concatenates the EW-MPFI exogenous features and the three-stage decomposition sub-signals along the channel dimension as variable tokens, so that the outputs of front-end selection and decomposition are directly fed into the cross-variable self-attention computation. In terms of multi-scale characterization, the cascade of signal decomposition and attention mechanisms has been shown to improve ultra-short-term wind power forecasting accuracy [47], yet the sub-components of different frequency bands are typically fed into a unified architecture, without identifying the differentiated modeling-capacity requirements of sub-components with distinct complexity levels. The MNE in this study assigns the high-, medium-, and low-frequency sub-components of the decomposition output to structurally differentiated parallel encoding branches, forming an end-to-end coupling with the front-end frequency-band decomposition and allowing the backbone to focus on local modeling of pre-aligned sub-scales. At a marginal contribution level of approximately 5%, these two components jointly play the key role of organizing the structural information from the front end and unifying the cross-variable and multi-scale pathways.

The design objective of probabilistic forecasting is to preserve the advantage in point-forecasting accuracy while achieving a reliable characterization of the predictive distribution. The non-crossing multi-output quantile regression network by Zhu et al., constrained by an end-to-end framework, exhibits a mutual trade-off between point accuracy and distributional characterization [22]. Even with a two-stage structure, the scheme by Zhang et al. uses neural networks to directly output distributional parameters without introducing point-forecasting anchors, requiring the probabilistic layer to relearn at full scale [48]. The NGBoost residual layer adopted in this study models the residual distribution on top of the three-quantile output of DPC-Former, achieving module-level decoupling between point accuracy and distributional characterization. At the ensemble level, the ensemble deep-learning non-crossing quantile regression by Cui et al. verified the benefit of ensembling for probabilistic forecasting, though at the cost of a heavy multi-stage training burden [49]. The NGBoost residual layer in this study decouples point-accuracy and distributional-characterization optimization through a two-stage structure, and parallel ensembling further completes the epistemic uncertainty, raising the PICP_80_ from 0.79 to 0.84 and delivering a reliable distributional characterization while preserving point accuracy. The horizons addressed here span 15 min to 4 h; extension to day-ahead scales and to minute-level ultra-short-term forecasting, each of which places different demands on the probabilistic layer, is left to future work.

These boundaries define the directions for future work. The single-site scope motivates cross-terrain and cross-season transfer learning; the quantified sensitivity of the offline decomposition motivates a strictly causal rolling-window, expanding-window, or online incremental implementation; and the present horizon range motivates extension toward both day-ahead and ultra-short-term scales. A further direction is the integration of the probabilistic forecasts into grid-dispatch reserve-capacity decisions.

## 6. Conclusions

To address the insufficient accuracy and reliability of multi-horizon probabilistic power forecasting for mountainous wind farms, this study develops an integrated forecasting framework. The framework consists of three core modules: EW-MPFI feature selection, three-stage signal decomposition, and the DPC-Former backbone together with the NGBoost probabilistic layer. The framework is validated on field measurements from a mountainous wind farm in Sichuan, China. The main conclusions are as follows.

(1) EW-MPFI selection reduces the test-set NMAE by 24.57% compared with the scheme that retains the 23-dimensional original inputs. The standard deviations of the three-perspective weights remain below 0.025 across 50 Bootstrap resamples, verifying the effectiveness of the method in alleviating the redundancy of multi-source meteorological inputs and the bias of any single importance metric, as well as the stability of the resulting ranking.
(2) The three-stage decomposition strategy reduces the test-set NMAE by 25.36% compared with the no-decomposition baseline and further outperforms four single-stage decomposition methods (SSA, CEEMDAN, VMD, and WPD) by 12% to 16%. Internal ablation shows that removing the SSA secondary refinement raises the NMAE by 6.33%, and further removing the multivariate entropy-weighted reconstruction raises it by a cumulative 10.94%, verifying the step-by-step mitigation of multi-scale modeling conflicts achieved by the three-stage strategy.
(3) The DPC-Former backbone and the NGBoost residual probabilistic layer reduce the test-set NMAE, NRMSE, pinball loss, and NCRPS by 3% to 35% compared with the deep-learning baselines and by approximately 70% compared with the ARIMA statistical baseline, with the PICP_80_ reaching 0.84 in close alignment with the nominal confidence level. The 50-seed ensemble further reduces NCRPS, NMAE, and NRMSE by 7.40%, 7.00%, and 13.70%, respectively, compared with the single-seed setting, verifying the decoupled optimization of point-forecasting accuracy and probabilistic distributional characterization enabled by the two-stage structure.
(4) The decomposition-window diagnostic indicates that the reported metrics represent offline best-case performance; strictly causal operational performance remains to be established through rolling- or expanding-window evaluation.

## Figures and Tables

**Table 1:** List of input variables for the model.

Variable	Measurement Height	Unit
Wind power	Plant-level	MW
Wind speed	10 m, 100 m	m/s
Wind direction	10 m, 100 m	°
Temperature	10 m	°C
Relative humidity	10 m	%
Atmospheric pressure	10 m	hPa

**Table 2:** Complexity metrics and reconstruction weights of each IMF computed on the training partition.

Component	PE	SE	Weight	Group
IMF1	0.999	0.379	0.012	HF
IMF2	0.884	0.340	1.988	HF
IMF3	0.710	0.288	0.662	MF
IMF4	0.581	0.331	0.955	MF
IMF5	0.502	0.265	1.136	MF
IMF6	0.453	0.166	1.247	MF
IMF7	0.419	0.059	0.938	LF
IMF8	0.405	0.021	0.961	LF
IMF9	0.395	0.013	0.977	LF
IMF10	0.389	0.005	0.987	LF
RES	0.295	0.001	1.138	LF

**Table 3:** Summary of the DPC-Former training protocol.

Item	Value
LOOKBACK	96 steps (24 h)
HIDDEN_DIM	64
NUM_LAYERS	2
NUM_HEADS	4
PATCH_LEN/PATCH_STRIDE	16/8
LEARNING_RATE	7.63 × 10^−4^
WEIGHT_DECAY	2.92 × 10^−6^
DROPOUT	0.348
LAMBDA_STAB	3.75 × 10^−5^
GRAD_CLIP	1.0
BATCH_SIZE	64
NUM_EPOCHS	100
EARLY_STOP_PATIENCE	30
SEED	50

**Table 4:** Horizon-wise performance of DPC-Former on the test set.

Horizon	NMAE (%)	NRMSE (%)	Pinball (%)	Cov80	Width80 (%)
15 min	2.56	4.11	0.88	0.81	8.25
1 h	4.78	6.85	1.55	0.76	14.53
4 h	8.27	11.18	2.64	0.74	23.77
AVG	5.21	7.38	1.69	0.77	15.52

**Table 5:** Horizon-wise performance of the NGBoost residual probabilistic layer.

Horizon	NCRPS (%)	NRMSE (%)	NMAE (%)	PICP_80_	PICP_90_	PINAW_80_ (%)	zstd
15 min	1.96	4.17	2.59	0.83	0.92	9.35	0.98
1 h	3.48	6.93	4.82	0.83	0.93	16.79	0.95
4 h	5.77	11.04	8.22	0.85	0.94	28.70	0.90
AVG	3.74	7.38	5.21	0.84	0.93	18.28	0.95

**Table 6:** Performance comparison of ablation variants for data preprocessing, feature engineering, and signal decomposition.

Variant	Configuration	NMAE (%)	NRMSE (%)	Δ NMAE
I-P-S	ICEEMDAN-PE&SE-SSA	5.21	7.38	—
I-P	Three-stage *w*/*o* SSA	5.54	7.89	+6.33%
ICEEMDAN	Single ICEEMDAN	5.78	8.03	+10.94%
SSA	Single SSA	5.85	8.09	+12.28%
CEEMDAN	Single CEEMDAN	6.00	8.26	+15.16%
VMD	Single VMD	6.03	8.49	+15.74%
WPD	Single WPD	5.97	8.16	+14.59%
NoDecomp	8-dim EW-MPFI only	6.53	9.50	+25.36%
NoPreprocess	Raw input	6.25	8.96	+19.96%
NoFeature	Full 23-dim	6.49	9.03	+24.57%

**Table 7:** Baseline model comparison.

Model	Params	NMAE (%)	NRMSE (%)	Pinball (%)	NCRPS (%)	PICP_80_
DPC-Former	0.34 M	5.21	7.38	1.69	3.74	0.84
ARIMA	—	19.20	23.15	7.66	5.16	0.89
LSTM	0.21 M	6.59	9.25	2.09	4.71	0.85
PatchTST	0.59 M	8.04	10.77	2.70	5.43	0.85
iTransformer	0.43 M	5.97	8.12	1.95	4.21	0.90
Transformer	0.41 M	5.42	7.60	1.79	3.94	0.83

**Table 8:** Multi-seed robustness statistics of DPC-Former.

Metric	Single-Seed Mean ± 95% CI	Ensemble Result	Ensemble Improvement
NCRPS (%)	4.04 ± 0.09	3.74	−7.40%
NMAE (%)	5.60 ± 0.12	5.21	−7.00%
NRMSE (%)	8.55 ± 0.21	7.38	−13.70%
Pinball (%)	1.86 ± 0.05	1.69	−9.20%
PICP_80_	0.79 ± 0.02	0.84	Mild widening (conservative)

**Table 9:** Performance comparison of alternative probabilistic-layer configurations.

Variant	Description	NMAE (%)	NRMSE (%)	Pinball (%)	NCRPS (%)	PICP_80_	zstd
V1	NGBoost	5.21	7.38	1.69	3.74	0.84	0.95
V2	Quantile	5.29	7.46	1.69	3.81	0.78	1.14
V3	QR	5.62	7.80	1.82	4.00	0.74	1.52
V4	KDE	5.69	7.91	1.96	4.28	0.75	1.29
V5	BNN	6.23	8.41	2.13	4.71	0.90	0.79
V6	MCM	5.73	8.17	1.96	4.28	0.69	1.52

**Table 10:** Internal component ablation of DPC-Former. “√” indicates that the corresponding component is retained, whereas “×” indicates that it is removed.

ID	Variant	Optuna	StabGate	CVA	MNE	NMAE (%)	NRMSE (%)	ΔNMAE
M1	DPC-Former	√	√	√	√	5.21	7.38	—
M2	M1 − Optuna	×	√	√	√	5.26	7.51	+0.96%
M3	M1 − StabGate	√	×	√	√	5.28	7.54	+1.34%
M4	M1 − CVA	√	√	×	√	5.45	7.80	+4.61%
M5	M1 − MNE	√	√	√	×	5.39	7.69	+3.45%

**Table 11:** Computational cost comparison of mainstream methods.

Model	Params	Train per Seed (min)	Inference
ARIMA	—	~50	~100
LSTM	0.21 M	0.18	0.04
iTransformer	0.43 M	0.28	0.03
Transformer	0.41 M	0.47	0.03
PatchTST	0.59 M	1.77	0.13
DPC-Former	0.34 M	0.33	0.04

**Table 12:** Effect of the decomposition fitting protocol on test-set accuracy. L0 is the partition-wise implementation used throughout this work; L1 is a control in which the decomposition is fitted over the entire record.

Horizon	NMAE (%) L0	NMAE (%) L1	Difference	Relative	NRMSE (%) L0	NRMSE (%) L1	NCRPS (%) L0	NCRPS (%) L1
15 min	2.689 ± 0.124	1.950 ± 0.073	0.739 pp	27.5%	4.248 ± 0.113	3.101 ± 0.114	1.846 ± 0.066	1.290 ± 0.051
1 h	5.263 ± 0.403	2.723 ± 0.123	2.540 pp	48.3%	7.583 ± 0.634	3.815 ± 0.204	3.492 ± 0.325	1.715 ± 0.056
4 h	8.804 ± 0.324	7.074 ± 0.090	1.730 pp	19.7%	12.142 ± 0.468	9.468 ± 0.193	5.849 ± 0.358	4.745 ± 0.135
Mean	5.585	3.916	1.670 pp	29.9%	7.991	5.461	3.729	2.583

**Table 13:** Sensitivity of test performance to the test-block decomposition-window length. The values are obtained from a fixed single-seed DPC-Former checkpoint and are used as a diagnostic rather than as replacements for the full-pipeline metrics.

Decomposition Window	ICEEMDAN Modes	NMAE (%)	ΔNMAE (pp)	NRMSE (%)	Pinball (%)	PICP_80_	PINAW_80_ (%)
Complete test block	10	5.733	—	8.174	1.960	0.678	12.771
Approximately weekly	8–9	5.733	+0.001	8.111	1.948	0.675	13.415
3 days	6–7	5.883	+0.150	8.285	2.001	0.679	13.919
1 day	5–6	6.684	+0.951	9.421	2.378	0.603	12.063

## Data Availability

The wind power dataset used in this study was provided by a third-party enterprise under a confidentiality agreement and is not publicly available due to commercial restrictions. The source code and processed data are available from the corresponding author upon reasonable request.
